# Antiplasmodial potential of isolated xanthones from *Mesua ferrea* Linn. roots: an in vitro and in silico molecular docking and pharmacokinetics study

**DOI:** 10.1186/s12906-024-04580-5

**Published:** 2024-07-25

**Authors:** Atthaphon Konyanee, Prapaporn Chaniad, Arnon Chukaew, Apirak Payaka, Abdi Wira Septama, Arisara Phuwajaroanpong, Walaiporn Plirat, Chuchard Punsawad

**Affiliations:** 1 College of Graduate Studies, Walailak University, Nakhon Si Thammarat, 80160 Thailand; 2 School of Medicine, Walailak University, Nakhon Si Thammarat, 80160 Thailand; 3 Research Center in Tropical Pathobiology, Walailak University, Nakhon Si Thammarat, 80160 Thailand; 4 Chemistry Department, Faculty of Science and Technology, Suratthani Rajabhat University, Surat Thani, 84100 Thailand; 5 School of Science, Walailak University, Nakhon Si Thammarat, 80160 Thailand; 6 Research Center for Pharmaceutical Ingredient and Traditional Medicine, Cibinong Science Center, National Research and Innovation Agency (BRIN), West Java, 16915 Indonesia

**Keywords:** Antiplasmodial activity, *Mesua ferrea* Linn., Xanthones, *Plasmodium falciparum*, Molecular docking, In silico pharmacokinetics

## Abstract

**Background:**

Malaria is a major global health concern, particularly in tropical and subtropical countries. With growing resistance to first-line treatment with artemisinin, there is an urgent need to discover novel antimalarial drugs. *Mesua ferrea* Linn., a plant used in traditional medicine for various purposes, has previously been investigated by our research group for its cytotoxic properties. The objective of this study was to explore the compounds isolated from *M. ferrea* with regards to their potential antiplasmodial activity, their interaction with *Plasmodium falciparum* lactate dehydrogenase (*Pf*LDH), a crucial enzyme for parasite survival, and their pharmacokinetic and toxicity profiles.

**Methods:**

The isolated compounds were assessed for in vitro antiplasmodial activity against a multidrug-resistant strain of *P. falciparum* K1 using a parasite lactate dehydrogenase (pLDH) assay. In vitro cytotoxicity against Vero cells was determined using the MTT (3-(4,5**-**dimethylthiazol-2-yl)-2,5**-**diphenyltetrazolium bromide) assay. The interactions between the isolated compounds and the target enzyme *Pf*LDH were investigated using molecular docking. Additionally, pharmacokinetic and toxicity properties were estimated using online web tools SwissADME and ProTox-II, respectively.

**Results:**

Among the seven compounds isolated from *M. ferrea* roots, rheediachromenoxanthone **(5)**, which belongs to the pyranoxanthone class, demonstrated good in vitro antiplasmodial activity, with the IC_50_ being 19.93 µM. Additionally, there was no toxicity towards Vero cells (CC_50_ = 112.34 µM) and a selectivity index (SI) of 5.64. Molecular docking analysis revealed that compound **(5)** exhibited a strong binding affinity of − 8.6 kcal/mol towards *Pf*LDH and was stabilized by forming hydrogen bonds with key amino acid residues, including ASP53, TYR85, and GLU122. Pharmacokinetic predictions indicated that compound **(5)** possessed favorable drug-like properties and desired pharmacokinetic characteristics. These include high absorption in the gastrointestinal tract, classification as a non-substrate of permeability glycoprotein (P-gp), non-inhibition of CYP2C19, ease of synthesis, a high predicted LD_50_ value of 4,000 mg/kg, and importantly, non-hepatotoxic, non-carcinogenic, and non-cytotoxic effects.

**Conclusions:**

This study demonstrated that compounds isolated from *M. ferrea* exhibit activity against *P. falciparum*. Rheediachromenoxanthone has significant potential as a scaffold for the development of potent antimalarial drugs.

**Supplementary Information:**

The online version contains supplementary material available at 10.1186/s12906-024-04580-5.

## Background

Malaria continues to be a global health issue affecting populations in many tropical and subtropical countries [1]. According to the World Malaria Report 2022, there were 247 million cases of malaria and 619,000 fatalities from the illness in 2021. The African region had the highest burden, accounting for approximately 95% of cases and 96% of global deaths [2]. Among the five *Plasmodium* species that cause malaria in humans, *P. falciparum* is the most lethal, accounting for over 90% of malaria-related fatalities worldwide [3].

In areas with endemic transmission, artemisinin-based combination therapies (ACTs) are recommended as first-line therapy for uncomplicated *P. falciparum* [4, 5]. ACTs involve a combination of artemisinin derivatives, which are fast-acting drugs capable of clearing most parasites within 3 days of treatment. These drugs are coupled with long-acting partner drugs to eliminate any remaining parasites [6]. Unfortunately, the emergence and spread of artemisinin resistance, particularly in the Greater Mekong Subregion (GMS), poses a serious threat to malaria control [7]. This resistance, characterized by delayed parasite clearance, has spread to other regions, including South America, Papua New Guinea, and Eastern Africa [8, 9]. Treatment efficacy heavily relies on the effectiveness of partner drugs, with resistance to drugs like mefloquine and piperaquine leading to high ACTs treatment failure rates [10, 11]. Therefore, the ongoing pursuit of novel antimalarial drugs remains an urgent priority for malaria control.

Medicinal plants play a crucial role in human well-being, serving as primary sources of medicines and reservoirs of phytochemicals that provide a foundation for the development of novel drugs [12]. Notably, 25% of new molecular entities (NMEs) approved by the Food and Drug Administration (FDA) are plant-based natural products [13]. Successful plant-derived antimalarial drugs include quinine, an alkaloid from *Cinchona* tree that serves as a scaffold for chloroquine synthesis, and artemisinin, a sesquiterpene lactone from *Artemisia annua* L. [14, 15]. Semi-synthetic derivatives of artemisinin, such as artesunate, dihydroartemisinin, and artemether, have been developed to improve bioavailability [16, 17]. The success of these plant-derived antimalarial drugs has led to the search for novel plant-based alternatives to address growing resistance to current treatments.

*Mesua ferrea* Linn., also known as Ceylon ironwood, belongs to the Calophyllaceae family. This tropical tree is distributed in most Southeast Asian countries, including India, Sri Lanka, and Thailand [18]. Whole plant parts of *M. ferrea* have been extensively utilized in traditional medicine for the treatment of various diseases. *M. ferrea* is traditionally used as a carminative, expectorant, diuretic, cardiotonic, antipyretic, and antimicrobial agent [19]. Several studies have demonstrated that phytochemicals derived from this plant possess a range of pharmacological properties, including antioxidant [20], anti-inflammatory [21], anti-microbial [22], antimalarial [23], anti-diabetic [24], anti-cancer [25], and immunomodulatory [19, 26]. Secondary metabolites isolated from *Mesua* species predominantly comprise xanthones and coumarins [27].

In a previous investigation, our research team effectively extracted bioactive xanthones from the roots of *M. ferrea* and investigated their cytotoxic effects against various cell lines, including A375 (human melanoma cells), PC-3 (human prostate cancer cells), and HaCaT (human keratinocyte immortal cells) [28]. However, to date, no further investigations have reported the antiplasmodial activity of compounds derived from *M. ferrea* roots. Therefore, the primary objective of the present study was to evaluate the antiplasmodial activities of selected xanthones isolated from *M. ferrea* roots. Furthermore, we also investigated their potential modes of action on the target *Plasmodium falciparum* lactate dehydrogenase (*Pf*LDH) through molecular docking techniques and integrated in silico pharmacokinetics and toxicity properties (ADMET) predictions into our methodology.

## Methods

### Parasite culture and maintenance

The *P. falciparum* K1 strain, kindly donated by Dr. Rapatbhorn Patrapuvich of Mahidol University, Thailand, was cultured in accordance with Trager and Jensen’s method, with minor modifications [29]. Briefly, the process involved thawing the cryopreserved parasites in a sodium chloride solution, following which the thawed parasites were transferred to Roswell Park Memorial Institute 1640 medium (Gibco, Carlsbad, CA, USA). The culture medium was supplemented with human type O-positive erythrocytes, 2 mg/mL of sodium bicarbonate (Sigma-Aldrich, St. Louis, MO, USA), 4.8 mg/mL of HEPES (C_8_H_18_N_2_O_4_S) (HiMedia, Mumbai, India), 10 µg/mL of hypoxanthine (C_5_H_4_N_4_O) (Sigma-Aldrich, USA), 0.5% of AlbuMAX™ II (a lipid-rich bovine serum albumin) (Gibco, Auckland, New Zealand), and 2.5 µg/mL of gentamycin (Sigma-Aldrich, New Delhi, India). The cultured parasites were incubated at 37 °C in a 5% CO_2_ atmosphere following an established protocol [30]. The percentage of parasitemia was determined by preparing thin blood smears, stained with Giemsa (Biotechnical, Bangkok, Thailand), and examined under a light microscope with a 100X oil immersion objective lens (Olympus CX31, Model CX31RBSFA, Tokyo, Japan).

### Extraction and isolation of compounds from *M. ferrea*

*M. ferrea* roots were collected from Trang province, Thailand (GPS coordinates: N7°18.781′, E99° 50.364′), and botanical identification was confirmed by an expert affiliated with the Forest Herbarium and assigned the voucher specimen number BKF 194350. Plant specimens were formally deposited at the Forest Herbarium and housed in the Department of National Parks, Wildlife, and Plant Conservation in Bangkok, Thailand.

In accordance with a previous study conducted by our research group, 8.3 kg of air-dried and pulverized *M. ferrea* L. root was sequentially extracted with 25 L of dichloromethane (CH_2_Cl_2_) and acetone solvents for a week at room temperature. The CH_2_Cl_2_ extract yielded 112.7 g of a yellow viscous residue, while the acetone extract yielded 25.0 g of a brownish residue. Seven compounds (1–7) were isolated from the CH_2_Cl_2_ and acetone extracts using chromatographic techniques. The compound structures were elucidated using spectroscopic methods [28]. Briefly, CH_2_Cl_2_ extract was subjected to quick column chromatography (QCC) on silica gel. Solvents with increasing polarities, *n*-hexane through ethyl acetate (EtOAc), were used for this purpose. The resulting eluates were combined into 12 fractions and subjected to thin-layer chromatography (TLC) analysis. Fraction 3 was subjected to column chromatography (CC) using an ethyl acetate (EtOAc)–hexane eluent (1:5, v/v), resulting in two compounds: 1-hydroxy-7-methoxyxanthone **(1)** and 1-hydroxy-5-methoxyxanthone **(2)**. Subsequently, eight subfractions (3A–3H) were obtained from this fraction. Subfraction 3H was further separated by CC using CH_2_Cl_2_–hexane (3.5:1.5, v/v) as the eluent to isolate 1,6-dihydroxyxanthone **(3)**. Fraction 4 was subjected to QCC using a solvent gradient from hexane to CH_2_Cl_2_ to methanol (MeOH). This process generated 12 sub-fractions (4A–4L). Compound **(4)**, 1,5-dihydroxyxanthone, was isolated from subfraction 4C *via* recrystallization using EtOAc-hexane (1:4, v/v). Fraction 5 was subjected to QCC with an EtOAc-hexane gradient, yielding five subfractions (5A–5E). Subfraction 5C was further separated by CC, employing an CH_2_Cl_2_–hexane eluent (4:2, v/v), resulting in six subfractions (5C1–5C6). Subfraction 5C6 was subsequently purified by TLC using EtOAc–hexane (1:5, v/v) as the eluent, leading to the isolation of rheediachromenoxanthone **(5)**. Fraction 7 was isolated using CC with a CH_2_Cl_2_–hexane eluent (4:2, v/v), yielding six subfractions (7A–7F). Subfraction 7E was further separated by CC, employing acetone–hexane elution (1:4, v/v), resulting in three subfractions (7E1–7E3). Sub-fraction 7E3 was subsequently purified by CC using EtOAc–hexane (1:4, v/v) as the elution solvent, leading to the isolation of 1,5-dihydroxy-3-methoxyxanthone **(6)**. The acetone extract was purified using QCC with hexane as the eluent, and the polarity was gradually increased from acetone to MeOH, resulting in six fractions. Fraction 4 was further purified by CC with an EtOAc–hexane eluent (2:3, v/v), yielding five subfractions (4A–4E). Sub-fraction 4D was purified by CC using EtOAc–hexane (2:3, v/v), yielding 2,5-dihydroxy-1-methoxyxanthone **(7)**.

**Fig. 1:**
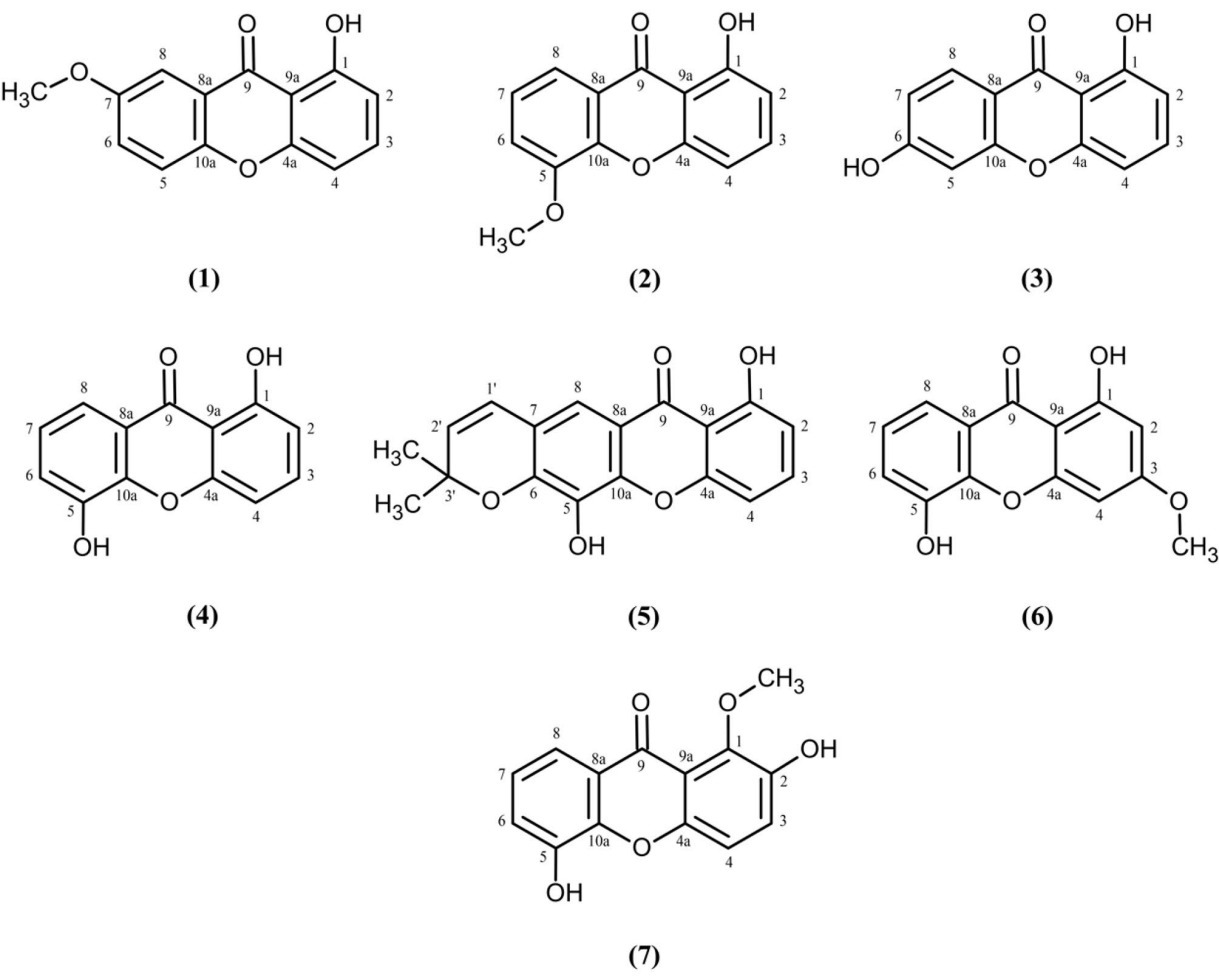
Chemical structure of compounds 1–7 isolated from *M. ferrea* L. roots

The chemical structures of all compounds were determined using NMR spectroscopic techniques. This structural determination was further corroborated by comparison with the available literature. In the present study, these compounds (Fig. 1) were evaluated for their antiplasmodial properties and selectivity.

### In vitro antiplasmodial activity

The objective of this study was to evaluate the antiplasmodial activity of the crude extracts and pure compounds isolated from *M. ferrea* L. roots. A modified parasite lactate dehydrogenase (pLDH) assay originally developed by Makler et al. (1993) was used [31]. Test extracts and pure compounds were dissolved in dimethyl sulfoxide (DMSO) (Merck, Darmstadt, Germany) to prepare stock solutions. Subsequently, the stock solutions were diluted to different concentrations ranging from 0.78 to 100 µg/mL. Two antimalarial drugs, artesunate and chloroquine (Sigma-Aldrich, USA), served as positive controls. DMSO and non-infected erythrocytes were used as the negative and baseline controls, respectively. Briefly, the infected erythrocytes (2% parasitemia and 2% erythrocytes) were transferred to a 96-well culture plate. Thereafter, the test extracts and pure compounds were added to each well in triplicate for each concentration. The plates containing the test samples were incubated at 37 °C in a 5% CO_2_ atmosphere for 72 h. Following incubation, the plates were subjected to three freeze-thaw cycles at − 20 °C, and 37 °C, to ensure erythrocyte lysis. The 20 µL of lysed erythrocyte solutions were then transferred to a new 96-well culture plate containing 100 µL/well of Malstat reagent (Merck) and 20 µL/well of *p*-nitroblue tetrazolium/phenazine ethosulfate (NBT/PES) solution (Sigma-Aldrich). The mixture was then incubated in the dark for 1 h. The absorbance of the resulting reactions was measured at 650 nm in a microplate reader following an established protocol [30]. The half-maximal inhibitory concentration (IC_50_) was calculated via nonlinear regression curve analysis using GraphPad Prism 6 (GraphPad Software, La Jolla, CA, USA).

### In vitro cytotoxicity assessment and selectivity index (SI)

The purpose of this study was to determine the cytotoxicity of crude extracts and pure compounds isolated from *M. ferrea* roots on Vero cells. The MTT assay was performed according to an established protocol [30]. Briefly, Vero cells were cultured in Dulbecco’s Modified Eagle Medium (Gibco, USA) supplemented with 10% fetal bovine serum (FBS) (Sigma-Aldrich, New Delhi, India). Vero cell suspensions were seeded onto 96**-**well culture plates at a density of 1 × 10^4^ cells per well. The seeded culture plates were incubated at 37 °C in a 5% CO_2_ atmosphere for 24 h to facilitate cell attachment. Subsequently, the cultured cells were exposed to the test extracts and pure compounds in triplicate for each concentration. Doxorubicin (Sigma-Aldrich, India) was used as a positive control, whereas DMSO served as a negative control. All extracts and pure compounds were dissolved in DMSO and added to the medium at a final concentration of 0.5% DMSO. Plates containing the test samples were incubated at 37 °C in a 5% CO_2_ atmosphere for 48 h. Following incubation, the cell culture supernatant was discarded, and 50 µL of MTT solution (5 mg/mL) (Thermo Fisher Scientific, Oregon, USA) was added to each well. The test plates were then incubated at 37 °C in a 5% CO_2_ atmosphere for 2 h. Subsequently, the supernatant was discarded, and 100 µL/well of DMSO was added to induce cell lysis and dissolve the formazan crystals. To estimate the reactions, absorbance was measured at 560 nm in a microplate reader, and the background absorbance at 670 nm was subtracted. The 50% cytotoxic concentration (CC_50_) was calculated using nonlinear regression curve analysis using GraphPad Prism 6. In addition, the selectivity index (SI) used to assess the ratio between the cytotoxicity against Vero cells (CC_50_) and the antiplasmodial activity against *P. falciparum* (IC_50_) was calculated for each crude extract and pure compound with the following formula below [32].

$$\:\text{S}\text{e}\text{l}\text{e}\text{c}\text{t}\text{i}\text{v}\text{i}\text{t}\text{y}\:\text{i}\text{n}\text{d}\text{e}\text{x}\:\left(\text{S}\text{I}\right)\:=\:{\text{C}\text{C}}_{50}/{\text{I}\text{C}}_{50}$$

### Molecular docking

#### Ligand preparation

The three-dimensional (3D) structures of the ligands were obtained from the PubChem database [33]. The ligands included in the study were 1-hydroxy-7-methoxyxanthone **(1)** (PubChem CID: 12214329), 1-hydroxy-5-methoxyxanthone **(2)** (PubChem CID: 86168207), 1,6-dihydroxyxanthone **(3)** (PubChem CID: 5493674), 1,5-dihydroxyxanthone **(4)** (PubChem CID: 5480299), rheediachromenoxanthone **(5)** (PubChem CID: 5488729), 1,5-dihydroxy-3-methoxyxanthone or Mesuaxanthone A **(6)** (PubChem CID: 5281651), 2,5-dihydroxy-1-methoxyxanthone **(7)** (PubChem CID: 10848779), artesunate (a standard antimalarial drug) (PubChem CID: 6917864), and chloroquine (a standard antimalarial drug) (PubChem CID: 2719).

To ensure stable ligand conformations, all the structures were subjected to geometry optimization and energy minimization. This was accomplished using conjugate gradient algorithms and a Universal Force Field [34], implemented using Avogadro software v. 1.2.0 [35]. Subsequently, Gasteiger charges were added, and nonpolar hydrogen atoms were merged using AutoDockTools (ADT) v. 1.5.7 [36], as described previously [37]. Finally, all ligands were saved in the Protein Data Bank (PDB) in Partial Charge (Q) and Atom Type (PDBQT) format.

#### Protein preparation

The X-ray crystallographic structure of the target protein *Pf*LDH complexed with 1,4-dihydronicotinamide adenine dinucleotide (NADH) and oxamate (PDB ID: 1LDG) [38] was obtained from the Research Collaboratory for Structural Bioinformatics Protein Data Bank (RCSB PDB) [39]. The resolution of the *Pf*LDH structure was determined to be 1.74 Å, which falls within the range of 1.5 to 2.5 Å for conducting high-quality molecular docking studies [40]. The *Pf*LDH structure was determined for subsequent molecular docking studies using ADT. This involved several steps, including adding missing residues and polar hydrogen atoms, removing water molecules and co-crystallized ligands, including NADH and oxamate, to create the apo form of *Pf*LDH, as described earlier [41]. The resulting *Pf*LDH structure was saved in the PDBQT format.

#### Molecular docking analysis

The potential molecular binding modes of the compounds isolated from *M. ferrea* roots, along with the antimalarial drugs chloroquine and artesunate, with the target enzyme *Pf*LDH were investigated using AutoDock Vina [42, 43]. Based on a previous study that identified amino acid residues that interact with NADH cofactors, including GLY29, MET30, ILE31, GLY32, ASP53, ILE54, TYR85, THR97, GLY99, PHE100, VAL138, ASN140, and HIS195, the active site of *Pf*LDH was determined [44]. Accordingly, a grid box was generated by ADT to cover the active site of *Pf*LDH, with the center coordinates set at center_x = 32 Å, center_y = 30 Å, and center_z = 32 Å, as described earlier [41, 45, 46]. Using ADT, the dimensions of the grid box were set to 26 × 26 × 26 Å^3^ for x, y, and z directions, with a grid spacing of 1.000 Å. Molecular docking simulations were performed using AutoDock Vina v. 1.1.2, with an exhaustiveness value of 24, while other parameters were maintained at their default settings. Compounds with a higher negative score indicated strong binding affinity and were selected as the most probable binding conformations [47]. To validate the docking protocol, the native ligand NADH was redocked into the active site of *Pf*LDH using the parameters mentioned earlier. Successful validation required the resulting root mean square deviation (RMSD) between the redocked ligand and the experimentally co-crystallized ligand to be below 2.0 Å. This ensured the reliability of the docking protocol before applying these parameters to other ligands [48, 49]. In this study, the RMSD of the redocked ligand was determined to be 0.775 Å, meeting the acceptance criteria of less than 2.0 Å, thereby affirming the accuracy and reliability of the docking protocol. Hydrogen bonds and hydrophobic interactions between the ligand atoms and amino acid residues of *Pf*LDH were identified using the Protein Ligand Interaction Profiler (PLIP) web tool, utilizing the default parameters [50, 51]. The protein-ligand complexes were rendered using the PyMOL molecular graphic system v. 2.5.2 (Schrödinger, LLC, New York, NY, USA).

### In silico pharmacokinetic and toxicity (ADMET) properties

The assessment of ADMET parameters is a crucial step in the early stages of drug testing and development, as this aims to reduce late-stage attrition in drug discovery programs [52]. In this context, we evaluated the ADMET properties of the most promising compounds in molecular docking studies in comparison with the antimalarial drugs chloroquine and artesunate. Towards this, we employed two online web tools: SwissADME [53] and ProTox-II [54]. In this process, all compounds under investigation were converted into their canonical Simplified Molecular Input Line Entry System (SMILES) format and subsequently submitted to SwissADME and ProTox-II. SwissADME was used to predict the physicochemical, drug-likeness, and pharmacokinetic properties. This tool also provides valuable parameters, including bioavailability radar plots and the Brain Or IntestinaL EstimateD (BOILED-Egg) predictive model. The assessment of drug likeness based on Lipinski’s rule of five (RO5) was used to determine the feasibility of a compound as an oral drug by considering four physicochemical parameters, as described in a previous review [55]. The bioavailability radar plot provides an initial overview of drug likeness through graphical representation by considering the optimal ranges of six key physicochemical properties, as described in SwissADME [53]. Moreover, the BOILED-Egg predictive model offered valuable insights into two pivotal pharmacokinetic parameters: passive human gastrointestinal absorption (HIA) and blood-brain barrier (BBB) permeation. In addition, this model facilitates the prediction of whether a compound functions as a substrate or non-substrate of P-glycoprotein (P-gp), a member of the ATP-binding cassette transporter (ABC transporters) [53, 56]. The fact that cytochrome P450 (CYP) enzymes play a significant role in drug elimination through metabolic biotransformation is essential and needs to be noted [53]. The inhibition of CYP enzymes is a major contributor to pharmacokinetic-related drug-drug interactions (DDIs), potentially leading to undesirable adverse effects [53]. Thus, the evaluation of compounds with the potential to inhibit CYP enzymes is a crucial aspect of drug discovery. Moreover, SwissADME offers a synthetic accessibility score ranging from 1 (indicating very easy synthesis) to 10 (indicating very difficult synthesis). Additionally, we employed the ProTox-II web server to predict various toxicological parameters, including acute oral toxicity, organ toxicity (hepatotoxicity), and various toxicological endpoints, including carcinogenicity, immunotoxicity, mutagenicity, and cytotoxicity, of the investigated compounds. Based on the globally harmonized system of classification and labeling of chemicals (GHS) using the LD_50_ thresholds described in ProTox-II, acute oral toxicity prediction was expressed the median lethal dose (LD_50_) in mg/kg and classified into six toxicity classes (class 1, toxic; class 6, non-toxic) [54, 57].

## Results

### In vitro antiplasmodial activity

The in vitro antiplasmodial activity against *P. falciparum* K1 of crude extracts and pure compounds isolated from *M. ferrea* L. roots is shown in Table 1. Antiplasmodial activity of crude extracts derived from plants is classified as follows: high activity (IC_50_ < 10 µg/mL), moderate activity (IC_50_ 11–50 µg/mL), low activity (IC_50_ 51–100 µg/mL), and inactive (IC_50_ > 100 µg/mL) [58]. According to these classifications, the dichloromethane extract of *M*. *ferrea* root exhibited moderate activity against the *P. falciparum* K1 strain, with an IC_50_ value of 13.63 µg/mL, whereas the acetone extract with an IC_50_ value exceeding 100 µg/mL had inactive antiplasmodial activity (Table 1). Similarly, the antiplasmodial activity of pure compounds derived from natural products is classified as follows: potent activity (IC_50_ < 1 µM), good activity (IC_50_ 1−20 µM), moderate activity (IC_50_ 20−100 µM), low activity (IC_50_ 100−200 µM), and inactive (IC_50_ > 200 µM) [59]. Based on this classification, among the seven compounds isolated from the roots, rheediachromenoxanthone **(5)** showed good antiplasmodial activity with an IC_50_ value of 19.93 µM, followed by 2,5-dihydroxy-1-methoxyxanthone **(7)**, which exhibited moderate activity with an IC_50_ value of 46.30 µM. While 1,5-dihydroxyxanthone **(4)**, 1-hydroxy-5-methoxyxanthone **(2)**, and 1,5-dihydroxy-3-methoxyxanthone **(6)** exhibited low activity, with IC_50_ values of 106.98, 163.75, and 198.27 µM, respectively, Conversely, 1,6-dihydroxyxanthone **(3)** and 1-hydroxy-7-methoxyxanthone **(1)** displayed inactive activity, with IC_50_ values of 226.13 and 345.56 µM, respectively (Table 1).

### In vitro cytotoxicity and SI values

The in vitro cytotoxicity against Vero cells of crude extracts and pure compounds isolated from *M. ferrea* L. roots is shown in Table 1. Crude extracts are classified as toxic to cells when the CC_50_ value is less than 30 µg/mL [60]. Similarly, pure compounds are considered toxic to cells when the CC_50_ value is less than 100 µM, respectively [61]. According to these classifications, the dichloromethane and acetone extracts exhibited non-toxic effects against Vero cells, with an CC_50_ value of 183.95 and > 800 µg/mL, respectively. Among the seven isolated compounds, **(1)**, **(3)**, **(4)**, **(5)**, **(6)**, and **(7)** demonstrated non-toxic effects against Vero cells, with CC_50_ values of 195.85, 182.78, 416.65, 112.34, 210.16, and 587.85 µM, respectively. In contrast, compound **(2)** exhibited toxic effects with a CC_50_ value of 62.01 µM (Table 1). Regarding the SI value, a higher SI value indicates more selectivity against *P. falciparum*. An SI value lower than 2 suggests general cytotoxicity, rendering the compound unsuitable for further development as an antimalarial candidate [62]. Based on this criterion, the dichloromethane extract exhibited moderate in vitro antiplasmodial activity with an SI value greater than 2, indicating selectivity against *P. falciparum* over Vero cells. Among the isolated compounds, compounds **(4)**, **(5)**, and **(7)** exhibited good to low in vitro antiplasmodial activity with SI values of 3.89, 5.64, and 12.70, respectively, indicating selectivity against *P. falciparum*. In contrast, compounds **(1)**, **(2)**, **(3)**, and **(6)** exhibited low to inactive in vitro antiplasmodial activity and SI values lower than 2, suggesting more selectivity towards Vero cells (Table 1).

**Table 1:** Antiplasmodial activity against the *P. falciparum* K1 strain and the cytotoxicity of crude extracts and compounds isolated from *M. ferrea* L. roots

Samples	IC_50_ ± SD (µM)	CC_50_ ± SD (µM)	Selectivity index (SI)
	*P*. *falciparum* K1	Vero cells	
Dichloromethane extract^c^	13.63 ± 0.26	183.95 ± 4.74	13.49
Acetone extract^c^	> 100	> 800	> 8
1-Hydroxy-7-methoxyxanthone **(1)**	345.56 ± 3.51^a, b^	195.85 ± 1.68^a, b^	0.57
1-Hydroxy-5-methoxyxanthone **(2)**	163.75 ± 1.71^a, b^	62.01 ± 1.18^a, b^	0.38
1,6-Dihydroxyxanthone **(3)**	226.13 ± 1.32^a, b^	182.78 ± 1.62^a, b^	0.81
1,5-Dihydroxyxanthone **(4)**	106.98 ± 4.41^a, b^	416.65 ± 1.98^a, b^	3.89
Rheediachromenoxanthone **(5)**	19.93 ± 0.72	112.34 ± 1.54^a, b^	5.64
1,5-Dihydroxy-3-methoxyxanthone **(6)**	198.27 ± 2.43^a, b^	210.16 ± 1.74^a.b^	1.06
2,5-Dihydroxy-1-methoxyxanthone **(7)**	46.30 ± 1.65^a, b^	587.85 ± 2.18^a, b^	12.70
Chloroquine	0.32 ± 0.00	ND	ND
Artesunate	0.003 ± 0.00	ND	ND
Doxorubicin	ND	3.35 ± 0.28	ND

ND: not determined

^a^ a statistically significant difference was observed between chloroquine and the test sample, ***p*** < 0.05 (mean ± SD of three determinations)

^b^ a statistically significant difference was observed between artesunate and the test sample, ***p*** < 0.05 (mean ± SD of three determinations)

^c^ IC_50_ unit of crude extracts expressed in µg/mL

### Molecular docking

**Fig. 2:**
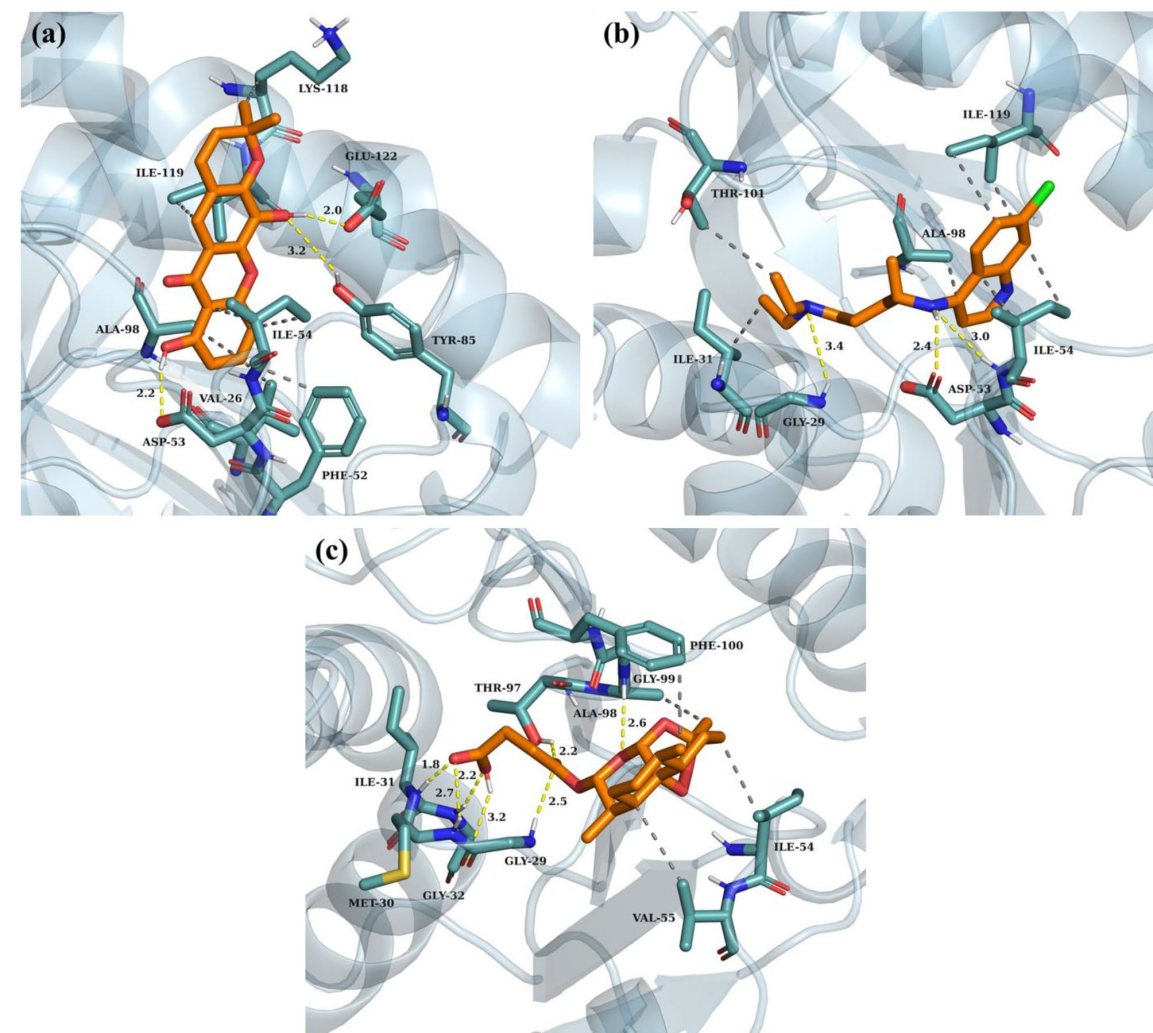
Predicted best binding mode of compounds within the active site of *Pf*LDH. The *Pf*LDH enzyme structure is represented as a light blue cartoon, and the interacting residues as ball and stick models, each labeled with its respective heteroatoms. The compounds are represented as ball and stick models and labeled according to their heteroatom elements: orange for carbon (C), white for hydrogen (H), red for oxygen (O), blue for nitrogen (N), and green for chlorine (Cl). Yellow dashed lines represent hydrogen bonds formed between interacting residues and compounds, with the associated bond length specified in angstroms (Å). Gray dashed lines represent hydrophobic interactions between interacting amino acid residues and the compounds. The compound, rheediachromenoxanthone (**a**), chloroquine (**b**), and artesunate (**c**)

**Fig. 3:**
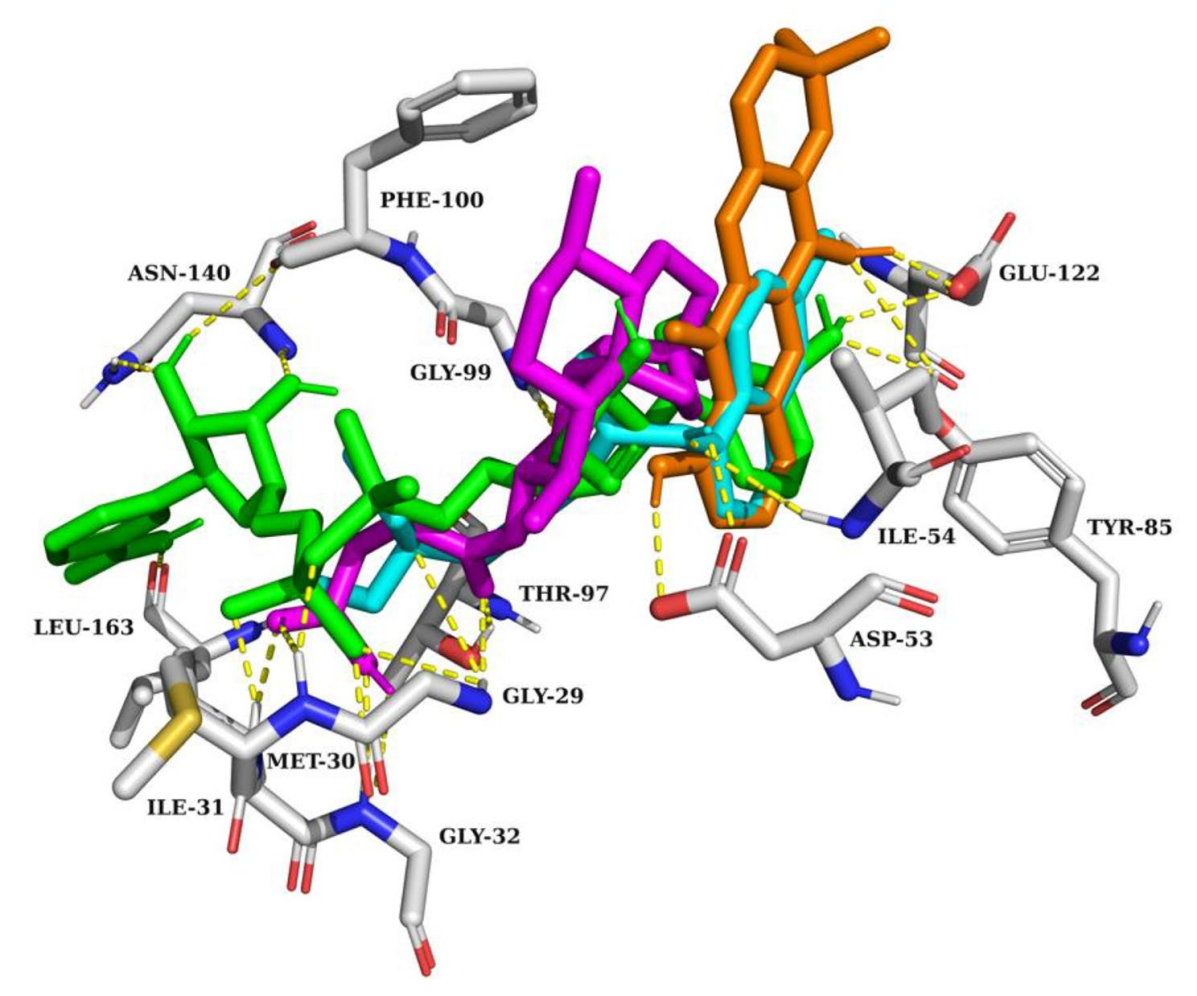
Superimposition of the best docked poses of rheediachromenoxanthone (orange), chloroquine (cyan), artesunate (magenta), and NADH (green) interacting with amino acid residues within the active site of *Pf*LDH. The yellow dashed lines represent the hydrogen bonds

A molecular docking study was performed to investigate the possible binding interactions between the compounds isolated from *M. ferrea* roots and the target protein, *Pf*LDH. The binding affinities and specific amino acid residues of *Pf*LDH involved in hydrogen bond and hydrophobic interactions with each compound are shown in Table 2. A high negative value of binding affinity indicates a strong interaction between the receptor and ligand. Among the seven compounds, rheediachromenoxanthone **(5)**, of the pyranoxanthone class, demonstrated the highest binding affinity of − 8.6 kcal/mol. The complex of rheediachromenoxanthone and *Pf*LDH was stabilized by three hydrogen bonds with amino acid residues, including ASP53 (2.2 Å), TYR85 (3.2 Å), and GLU122 (2.0 Å). In addition, the complex is stabilized by seven hydrophobic interactions with amino acid residues, including VAL26, PHE52, ILE54 (two interactions), ALA98, LYS118, and ILE119. The methyl group positioned on the pyran ring is responsible for the hydrophobic interactions with the amino acid residue LYS118 (Fig. 2a). Significantly, the compound structure exhibited a remarkable fit within the active site of *Pf*LDH, resulting in the formation of hydrogen bonds between the two hydroxyl groups at C1 and C5 with the same amino acid residues crucial for cofactor enzyme (NADH) binding (Fig. 3). Remarkably, rheediachromenoxanthone exhibited good in vitro antiplasmodial activity (IC_50_ = 19.93 µM), which strongly correlates with its exceptional binding affinity. Followed by artesunate, the potent antimalarial drug, which demonstrated a binding affinity slightly lower than rheediachromenoxanthone, with a binding affinity − 8.0 kcal/mol. Artesunate is stabilized by strong seven hydrogen bonds with amino acid residues, including GLY29 (2.5 Å and 3.2 Å), MET30 (2.7 Å), ILE31 (1.8 Å), GLY32 (2.2 Å), THR97 (2.2 Å), and GLY99 (2.6 Å), and by four hydrophobic interactions with amino acid residues, including ILE54, VAL55, ALA98, and PHE100, respectively (Fig. 2c). While chloroquine, the antimalarial drug, demonstrated a weak interaction with *Pf*LDH with a binding affinity − 7.2 kcal/mol. Chloroquine is stabilized by three hydrogen bonds with amino acid residues, including GLY29 (3.4 Å), ASP53 (2.4 Å), and ILE54 (3.0 Å), and by seven hydrophobic interactions with amino acid residues, including ILE31, ILE54 (two interactions), ALA98, THR101, and ILE119 (two interactions), respectively (Fig. 2b).

**Table 2:** Binding affinity and interacting amino acid residues of compounds isolated from *M. ferrea* L. roots with *Pf*LDH

Compound	Binding affinity (kcal/mol)	Hydrogen bond interaction			Hydrophobic interaction	
		Number of interactions	Amino acid residues		Number of interactions	Amino acid residues
1-Hydroxy-7-methoxyxanthone **(1)**	–7.5	1	TYR85	5		PHE52, ILE54^a^, ALA98, ILE119
1-Hydroxy-5-methoxyxanthone **(2)**	–7.6	1	GLU122	4		PHE52, ILE54, ALA98, ILE119
1,6-Dihydroxyxanthone **(3)**	–7.6	2	ASP53, ILE54	6		VAL26, PHE52, ILE54^a^, ALA98, ILE119
1,5-Dihydroxyxanthone **(4)**	–7.7	2	ILE54, GLU122	4		VAL26, PHE52, ILE54, ALA98
Rheediachromenoxanthone **(5)**	–8.6	3	ASP53, TYR85, GLU122	7		VAL26, PHE52, ILE54^a^, ALA98, LYS118, ILE119
1,5-Dihydroxy-3-methoxyxanthone **(6)**	–7.5	2	ASP53, ILE54	4		VAL26, PHE52, ILE54, ALA98
2,5-Dihydroxy-1-methoxyxanthone **(7)**	–7.4	2	ASP53, TYR85	4		PHE52, ILE54, ALA98, ILE119
Chloroquine	–7.2	3	GLY29, ASP53, ILE54	7		ILE31, ILE54^a^, ALA98, THR101, ILE119^a^
Artesunate	–8.0	7	GLY29^a^, MET30, ILE31, GLY32, THR97, GLY99	4		ILE54, VAL55, ALA98, PHE100

^a^Two interactions with amino acid residues.

In addition, 2,5-dihydroxy-1-methoxyxanthone **(7)** exhibited moderate in vitro antiplasmodial activity (IC_50_ = 46.30 µM) and a binding affinity of − 7.4 kcal/mol. Compound **(7)** was stabilized by two hydrogen bonds with amino acid residues ASP53 and TYR85 and by four hydrophobic interactions with amino acid residues PHE52, ILE54, ALA98, and ILE119 (Table 2). While, 1,5-dihydroxyxanthone **(4)** exhibited low in vitro antiplasmodial activity (IC_50_ = 106.98 µM) and a binding affinity of − 7.7 kcal/mol. Compound **(4)** was stabilized by two hydrogen bonds with amino acid residues ILE54 and GLU122 and by four hydrophobic interactions with amino acid residues VAL26, PHE52, ILE54, and ALA98 (Table 2). These findings are consistent with those observed for 1-hydroxy-5-methoxyxanthone **(2)** (IC_50_ = 163.75 µM) and 1,5-dihydroxy-3-methoxyxanthone **(6)** (IC_50_ = 198.27 µM). Compound **(2)** demonstrated a binding affinity of − 7.6 kcal/mol and was stabilized by only one hydrogen bond with the amino acid residue GLU122 and by four hydrophobic interactions with amino acid residues PHE52, ILE54, ALA98, and ILE119 (Table 2). Compound **(6)** demonstrated a binding affinity of − 7.5 kcal/mol and was stabilized by two hydrogen bonds with the amino acid residue ASP53 and ILE54 and by four hydrophobic interactions with amino acid residues VAL26, PHE52, ILE54, and ALA98 (Table 2). In particular, 1-hydroxy-7-methoxyxanthone **(1)**, and 1,6-dihydroxyxanthone **(3)** displayed inactive in vitro antiplasmodial activities, with IC_50_ values of 345.56 and 226.13 µM, respectively. Compounds **(1)** and **(3)** demonstrated binding affinity of − 7.5 and − 7.6 kcal/mol, respectively (Table 2). Based on the molecular docking results presented in this study, it is evident that compounds **(1)**, **(2)**, **(3)**, **(4)**, **(6)**, and **(7)** belonging to the simple oxygenated xanthone class exhibit slightly varying binding affinities, ranging from − 7.4 to − 7.7 kcal/mol. These binding affinities are in line with the observed in vitro antiplasmodial activity, which ranged from moderate to inactive. In contrast, rheediachromenoxanthone, classified as a pyranoxanthone, demonstrated the highest favorable binding affinity of − 8.6 kcal/mol, suggesting a strong propensity to effectively bind within the active site of *Pf*LDH, a characteristic that strongly aligns with its good in vitro antiplasmodial activity. These results highlight the potential impact of structural distinctions on their respective antiplasmodial mechanisms.

### In silico pharmacokinetic and toxicity (ADMET) properties

**Fig. 4:**
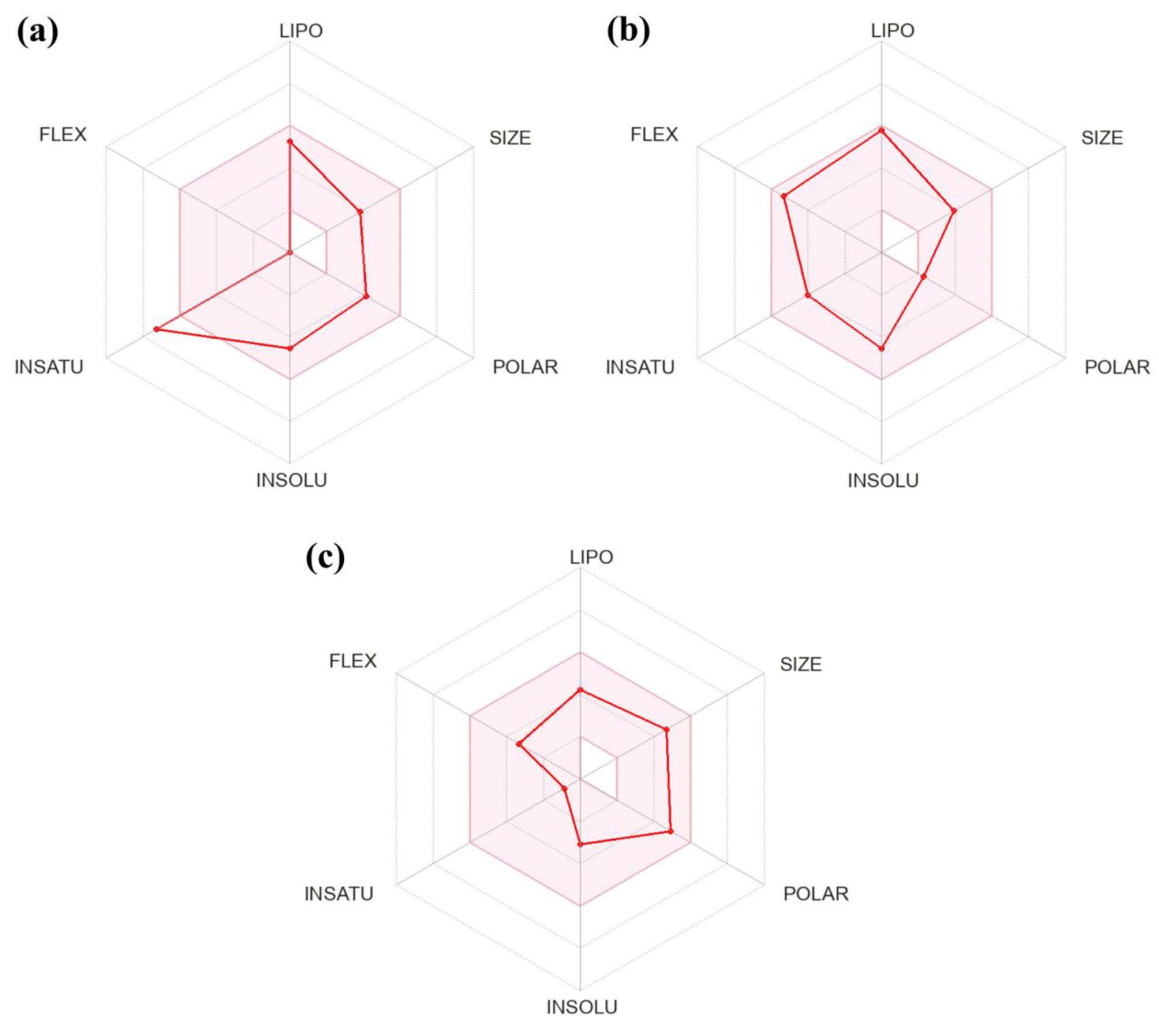
Bioavailability radar plot for rheediachromenoxanthone (**a**), chloroquine (**b**), and artesunate (**c**). The pink area represents the optimal range for six physicochemical properties associated with oral bioavailability, and the red line represents the specific properties of each compound

**Fig. 5:**
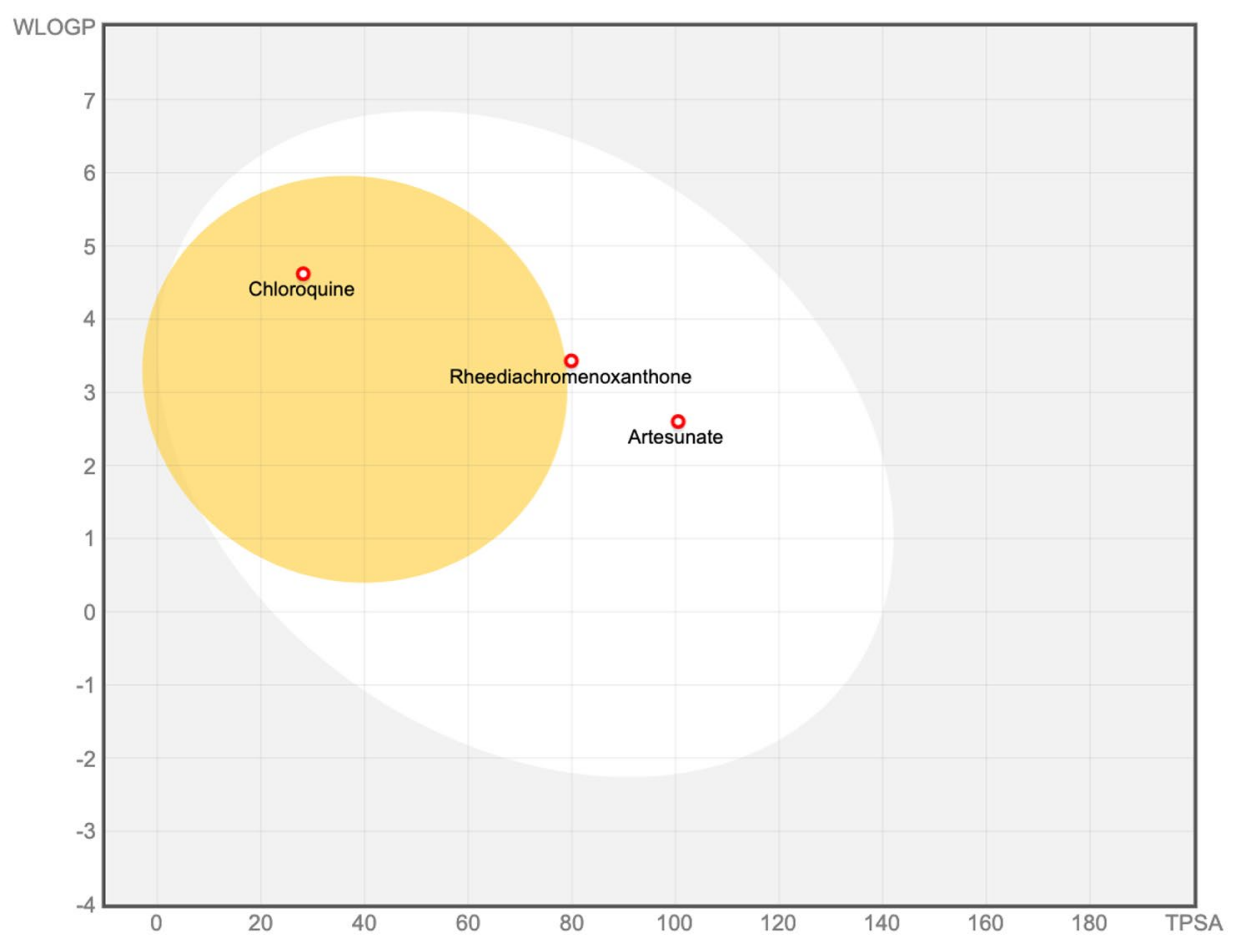
BOILED-Egg predictive model shows the placement of rheediachromenoxanthone, chloroquine, and artesunate. This model is conceptually grounded in two essential physicochemical properties: the WLOGP value, which reflects lipophilicity, and the TPSA value, which reflects polarity. The compound falling within the white region (albumin) represents a high probability of HIA absorption, while the compound falling within the yellow region (yolk) represents a high probability of BBB permeation. The compound falling within the gray region is unlikely to be absorbed through HIA or the BBB. The red dots represent the compound as the P-gp non-substrate (PGP-).

Based on the in vitro antiplasmodial activity and molecular docking, rheediachromenoxanthone was identified as the most promising compound. Consequently, this compound was selected for a further comprehensive investigation of its ADMET properties in comparison to the antimalarial drugs chloroquine and artesunate. The predicted physicochemical properties, drug-likeness, pharmacokinetic properties, and toxicity profiles of the investigated compounds are summarized in Table 3. The drug-likeness results demonstrated that none of the investigated compounds violated RO5, suggesting that all of them possess the potential to be considered for oral drugs (Table 3). The bioavailability radar considers six physicochemical parameters, with the pink areas representing the optimal ranges for each property. The results demonstrated that chloroquine and artesunate fell within the optimal range for all properties, thereby categorizing them as drug-like (Fig. 4b and c). Rheediachromenoxanthone demonstrated a slight deviation from the optimal range of saturation properties (fraction Csp^3^) (Fig. 4a). The BOILED-Egg predictive model revealed distinct patterns for the investigated compounds. Chloroquine was placed within the yellow region (yolk), indicating a high probability of BBB permeation. In contrast, rheediachromenoxanthone and artesunate were placed within the white region (albumin), indicating a pronounced likelihood of HIA. Furthermore, all compounds exhibited a non-substrate of P-gp (PGP–), indicating that they were not prone to efflux from either the central nervous system (CNS) or gastrointestinal tract (Fig. 5). The assessment of the inhibitory activity against the five major CYP isoforms by the investigated compounds revealed distinct patterns. Rheediachromenoxanthone inhibited CYP1A2, CYP2C9, CYP2D6, and CYP3A4, while chloroquine showed inhibitory activities against CYP1A2, CYP2D6, and CYP3A4. In contrast, artesunate did not inhibit any of the investigated CYP isoforms. Interestingly, none of the investigated compounds showed inhibitory activity against CYP2C19 (Table 3). The synthetic accessibility scores for rheediachromenoxanthone, chloroquine, and artesunate demonstrated varying degrees of ease of synthesis. Rheediachromenoxanthone, in particular, showed a low score of 3.57, implying relative ease of synthesis. Similarly, chloroquine had a low score of 2.76. In contrast, artesunate showed a notably higher score (6.67), indicating a significantly more complex and challenging synthetic procedure. In addition, the toxicity profiles, as determined through ProTox-II predictions, demonstrated that rheediachromenoxanthone was classified as toxicity class 5 for acute oral toxicity, with a predicted LD_50_ of 4,000 mg/kg. In contrast, chloroquine and artesunate were categorized as toxicity class 4 for acute oral toxicity, with predicted LD_50_ values of 750 mg/kg and 1,000 mg/kg, respectively. Moreover, all the investigated compounds showed no hepatotoxicity, carcinogenicity, or cytotoxicity. In contrast, all the investigated compounds exhibited immunogenicity. In addition, rheediachromenoxanthone and chloroquine exhibited active mutagenicity with prediction probabilities of 0.55 and 0.94, respectively (Table 3).

**Table 3:** Physicochemical properties, drug-likeness, pharmacokinetics properties, medicinal chemistry, and toxicity profiles of rheediachromenoxanthone, chloroquine, and artesunate

Type of parameters	Parameters	Compound		
		Rheediachromenoxanthone	Chloroquine	Artesunate
Physicochemical	MW (g/mol)	310.30	319.87	384.42
	Fraction Csp^3^	0.17	0.50	0.89
	Number of rotatable bonds	0	8	5
	Number of HBA	5	2	8
	Number of HBD	2	1	1
	Molar Refractivity	88.16	97.41	92.46
	TPSA (Å^2^)	79.90	28.16	100.52
Lipophilicity	Log *P*_o/w_ (iLOGP)	3.04	3.95	2.62
	Log *P*_o/w_ (XLOGP3)	3.73	4.63	1.88
	Log *P*_o/w_ (WLOGP)	3.43	4.62	2.60
	Log *P*_o/w_ (MLOGP)	1.48	3.20	1.88
	Log *P*_o/w_ (SILICOS-IT)	3.36	4.32	1.37
	Consensus Log *P*_o/w_	3.01	4.15	2.07
Water solubility	Log *S* (ESOL)	–4.56	–4.55	–3.08
Drug-likeness	Lipinski’s rule of five (RO5)	Yes; 0 violation	Yes; 0 violation	Yes; 0 violation
Pharmacokinetics	HIA absorption	High	High	High
	BBB permeant	No	Yes	No
	P-gp substrate	No	No	No
	CYP1A2 inhibitor	Yes	Yes	No
	CYP2C19 inhibitor	No	No	No
	CYP2C9 inhibitor	Yes	No	No
	CYP2D6 inhibitor	Yes	Yes	No
	CYP3A4 inhibitor	Yes	Yes	No
Medicinal chemistry	Synthesis accessibility score	3.57	2.76	6.67
Toxicity	Predicted LD_50_ (mg/kg)	4000	750	1000
	Toxicity class	5 (may be harmful)	4 (harmful)	4 (harmful)
	Hepatotoxicity (probability)	Inactive (0.80)	Inactive (0.90)	Inactive (0.76)
	Carcinogenicity (probability)	Inactive (0.53)	Inactive (0.66)	Inactive (0.65)
	Immunotoxicity (probability)	Active (0.99)	Active (0.99)	Active (0.87)
	Mutagenicity (probability)	Active (0.55)	Active (0.94)	Inactive (0.63)
	Cytotoxicity (probability)	Inactive (0.81)	Inactive (0.93)	Inactive (0.87)

## Discussion

In this study, all seven compounds isolated from the roots of *M. ferrea* were identified as xanthones, which are phenolic compounds characterized by a planar dibenzo-γ-pyron scaffold. Benzene rings A and B joined together by an oxygen atom and a carbonyl group (ring C), form the basic scaffold of xanthone [63]. Xanthones have been isolated from various natural sources, including higher plants, lichens, and microorganisms. These compounds possess various pharmacological properties, including anti-inflammatory, antioxidant, antimicrobial, and anticancer activities [64]. The interactions between xanthones and diverse pharmacological targets depend on the substitutions present on the two benzene rings [65]. The xanthones isolated from *Mesua* species are classified as three main groups of natural xanthones, including simple oxygenated xanthones, prenylated and related xanthones, and bisxanthones [27].

We investigated the antiplasmodial activities of dichloromethane and acetone extracts and compounds isolated from the roots of *M. ferrea* against the *P. falciparum* K1 strain. Among the seven compounds, rheediachromenoxanthone **(5)** exhibited the most substantial antiplasmodial activity against *P. falciparum* K1, with an IC_50_ value of 19.93 µM. However, rheediachromenoxanthone **(5)** exhibited lower antiplasmodial potency, being approximately 62.3 and 6,643.3-fold less potent than chloroquine and artesunate, respectively. In contrast, the simple oxygenated xanthone 2,5-dihydroxy-1-methoxyxanthone **(7)** displayed an IC_50_ value of 46.30 µM. Rheediachromenoxanthone **(5)** possessed markedly superior antiplasmodial activity, being approximately 2.3 times more potent than 2,5-dihydroxy-1-methoxyxanthone **(7)**. The remarkable antiplasmodial activity of rheediachromenoxanthone **(5)** can be attributed to its unique structural characteristics, including an additional linear dimethylpyran ring. Our findings are in accordance with a previous study, which demonstrated that caloxanthone C, a pyranoxanthone isolated from the root barks of *Calophyllum caledonicum* that contains an additional pyran ring, had enhanced antiplasmodial activity against the chloroquine-resistant strains of *P. falciparum* FcB1/Colombia with an IC_50_ value of 1.3 µg/mL, as compared to its derivative, 1,3,5-trihydroxyxanthones, which demonstrated a considerably higher IC_50_ value of 24.7 µg/mL [66]. Additionally, macluraxanthone, a pyranoxanthone isolated from the stem bark of *Garcinia bancana* Miq. that contains a linear dimethylpyran ring and dimethylallyl group, has shown potent antiplasmodial activity against chloroquine-sensitive strains of *P. falciparum* 3D7 with an IC_50_ value of 4.28 µM when compared to isojacareubin, which contains only an angular dimethylpyran ring and displayed a considerably higher IC_50_ value of 11.45 µM [67].

Simple oxygenated xanthones were also observed in the extracts of *M. ferrea* root. The results showed that 2,5-dihydroxy-1-methoxyxanthone **(7)**, which substitutes with two hydroxyl groups at C2 and C5 and one methoxy group at C1, demonstrated moderate antiplasmodial activity with an IC_50_ value of 46.30 µM. In contrast, 1,5-dihydroxyxanthone **(4)**, which substitutes two hydroxyl groups at C1 and C5, exhibited low antiplasmodial activity with an IC_50_ value of 106.98 µM. Furthermore, 1-hydroxy-5-methoxyxanthone **(2)**, which substitutes with one hydroxyl group at C1 and one methoxy group at C5, had an IC_50_ value of 163.75 µM. Similarly, 1,5-dihydroxy-3-methoxyxanthone **(6)**, which substitutes with two hydroxyl groups at C1 and C5 and one methoxy group at C3, exhibited an IC_50_ value of 198.27 µM. Conversely, 1,6-dihydroxyxanthone **(3)**, which substitutes for two hydroxyl groups at C1 and C6, demonstrated inactive antiplasmodial activity with an IC_50_ value of 226.13 µM. Additionally, 1-hydroxy-7-methoxyxanthone **(1)**, which substitutes with one hydroxyl group at C1 and one methoxy group at C7, demonstrated the lowest antiplasmodial activity with an IC_50_ value of 345.56 µM.

These results indicated that the position and number of hydroxyl groups in the simple oxygenated xanthone structure have a significant impact on their antiplasmodial activity. Compounds with two hydroxyl groups at specific positions, C2 and C5, tended to have enhanced antiplasmodial activity, whereas the presence of hydroxyl groups at C1 and C5 or C1 alone tended to reduce activity. However, the number and position of methoxy groups can have varying effects on antiplasmodial activity. Our findings are consistent with those of a previous study, which demonstrated that compound 2,3,4,5,6-pentahydroxyxanthone potently inhibited in vitro heme polymerization with an IC_50_ value of 1.2 µM. This observation was consistent with its in vitro antiplasmodial activity against the chloroquine-sensitive strain of *P. falciparum* D6, with an IC_50_ value of 0.4 µM. The study demonstrated that a high number of hydroxyl groups is advantageous for inhibitory activity. This underscores the significance of the hydroxyl groups at positions C4 and C5, as they play a pivotal role in interacting with heme carboxylate groups, consequently leading to the inhibition observed in the in vitro heme polymerization assay. Additionally, the same study found that 2,3,4,5,6-pentamethoxyxanthone displayed no in vitro antiplasmodial activity or heme polymerization [68]. In addition, previous study demonstrated the substitution of a methoxy group on xanthone structure did not significantly enhance antimalarial activity [69]. Another study demonstrated that the presence of a hydroxyl group at the periposition, specifically at C1 or C8, results in reduced antiplasmodial activity. For instance, 1,3,5-trihydroxyxanthone displayed inactivity against *P. falciparum* D6, with an IC_50_ value exceeding 60 µM [70]. Regarding the SI values, among the isolated compounds, rheediachromenoxanthone **(5)** exhibited an SI value greater than 2. This indicates good selectivity against *P. falciparum* compared to normal cells (Vero cells). Therefore, rheediachromenoxanthone **(5)** should be prioritized for further investigation and development as a potential antimalarial candidate.

Structure-based drug design (SBDD) is an integral component of computer-aided drug design (CADD). Several drugs, such as captopril (Bristol Myers Squibb), saquinavir (Roche), zanamivir (Biota), and imatinib (Novartis), have successfully reached the market through the application of the SBDD technique [71]. Among the various SBDD techniques, molecular docking is the most widely used, primarily because of its capacity to facilitate a comprehensive understanding and prediction of molecular recognition interactions between small molecules and target macromolecules [72].

*Pf*LDH serves as the terminal enzyme in the glycolytic pathway and is responsible for converting pyruvate to lactate using NADH as a cofactor. Among the LDH enzymes in human *Plasmodium* species (*P. vivax*, *P. malariae*, and *P. ovale*), in comparison to *Pf*LDH, the sequence identity ranges from 90 to 92% [44]. A previous study revealed that the frequency of amino acid substitutions in global *Pf*LDH populations is low, ranging from 0.3 to 3.3%. This observation suggests that the genetic polymorphism in *Pf*LDH is well conserved, making it a promising candidate for drug development [73]. Furthermore, *Pf*LDH exhibited a distinctive structural feature absent in human lactate dehydrogenase (hLDH), with the insertion of five amino acid residues within substrate-specific loops [74]. Additionally, *Pf*LDH demonstrates distinct enzyme kinetic properties compared with hLDH [44, 75]. Ideally, antimicrobial molecular targets are selected based on their complete absence from human hosts [76]. In line with this principle, we selected *Pf*LDH as the target owing to its distinctive structural and kinetic attributes relative to hLDH. The inhibition of *Pf*LDH reduces ATP production, ultimately leading to the killing of the parasite [77]. Previous studies have shown that the azole-based compounds inhibit *Pf*LDH activity, correlating with their efficacy against both chloroquine-sensitive and chloroquine-resistant *P. falciparum* (whole cells) [78]. For these reasons, several studies have proposed *Pf*LDH as a promising molecular target for the development of novel antimalarial agents [38, 44, 77].

Molecular docking showed that rheediachromenoxanthone **(5)** exhibited the highest binding affinity towards *Pf*LDH, with a binding affinity of − 8.6 kcal/mol. This high binding affinity is correlated with its good in vitro antiplasmodial activity, as evidenced by an IC_50_ value of 19.93 µM. In contrast, six simple oxygenated xanthones displayed binding affinities with *Pf*LDH ranging from − 7.4 to − 7.7 kcal/mol, which correlated with their in vitro antiplasmodial activities varying from moderate to inactive. Notably, hydroxyl groups present in the xanthone structure play a crucial role in forming hydrogen bonds with the amino acid residues within the active site of *Pf*LDH.

Chloroquine being previously identified as a weak inhibitor of *Pf*LDH [79]. From our results, chloroquine interacts with *Pf*LDH by forming three hydrogen bonds involving amino acid residues GLY29, ASP53, and ILE54. This observation aligns with a previous study that identified chloroquine binding to ASP53 and GLY99 of *Pf*LDH [80]. Additionally, the quinoline ring of chloroquine occupied a position similar to that of the adenine ring of the NADH cofactor, which is consistent with earlier studies that identified chloroquine as a competitive inhibitor of the NADH cofactor [44, 81].

Artesunate is currently used as a first-line treatment for *P. falciparum*. The mechanism of activation of artemisinin and its derivatives involves cleavage of the endoperoxide bridge by either ferrous iron or heme, a byproduct of hemoglobin degradation in the digestive vacuole of parasites. This cleavage process generates carbon-centered radicals that can alkylate several essential proteins within the parasite [82]. In this study, artesunate exhibited a strong interaction with *Pf*LDH with a binding affinity of − 8.0 kcal/mol compared to chloroquine. Previous studies have verified that *Pf*LDH is a key enzyme susceptible to covalent modification by the bulk of activated artemisinin, leading to the irreversible disruption of its enzymatic activity [83].

According to the docking results, ASP53, ILE54, TYR85, and GLU122 are crucial amino acid residues within the *Pf*LDH active site responsible for the interactions formed by hydrogen bonds with the isolated xanthone compounds. Our findings are similar to those of previous studies. The results demonstrated that compound 2, a derivative of 1-(heteroaryl)-2-((5-nitroheteroaryl)methylene) hydrazine, formed five hydrogen bonds with the amino acid residues within the active site of *Pf*LDH. These interactions involve ILE31, GLY32, ASP53, TYR85, and THR97. Remarkably, this binding pattern correlated with a high suppression of parasitemia, with a 99.09% suppression observed at a dose of 125 mg/kg body weight in Peter’s test [45]. In addition, synthetic quinoline-based compounds form hydrogen bonds with the amino acid residues within the active site of *Pf*LDH. These interactions involve ASP53, ILE54, and GLY99 [84]. Moreover, previous studies demonstrated that the DAQ compound (chloroquine analog) exhibited potent antimalarial activity in both in vitro and in vivo models, consistent with the in silico prediction that the DAQ exhibited favorable interaction with *Pf*LDH at the NADH binding site [81]. In line with these findings, our study demonstrated that rheediachromenoxanthone **(5)** exhibited good in vitro antiplasmodial activity and favorable interaction with specific amino acid residues within the active site of *Pf*LDH, including ASP53, TYR85, and GLU122. These compelling results strongly support the selection of rheediachromenoxanthone **(5)** for further development as a novel inhibitor targeted at *Pf*LDH. The molecular dynamics studies can be employed for further stability studies.

Regarding the other molecular targets of significant importance in the development of novel antimalarial drugs, such as *P. falciparum* dihydrofolate reductase-thymidylate synthase (*Pf*DHFR-TS), as a crucial molecular target for designing antifolate antimalarial drugs, inhibition of this enzyme reduces the production of deoxythymidine monophosphate (dTMP) and DNA base synthesis [85]. The cytochrome *bc*_1_ complex (Complex III) is a crucial enzyme in the electron transport chain of *P. falciparum*, which also helps with the reoxidation of ubiquinol, which is necessary for ubiquinone-dependent dihydroorotate dehydrogenase (DHODH), an enzyme essential for pyrimidine biosynthesis [86, 87]. Inhibition of this enzyme by a known antimalarial drug (atovaquone) disrupts the mitochondrial membrane potential (ΔΨm), leading to parasite death [88]. Moreover, DHODH is the fourth enzyme in the pyrimidine biosynthesis pathway and is essential for parasite survival because parasites cannot salvage pyrimidines [86]. The novel antimalarial agent triazolopyrimidine, DSM265, which targets DHODH, has successfully entered clinical trial [89].

ADMET properties serve as critical factors in explaining how candidate compounds achieve appropriate concentrations at the therapeutic site of action while simultaneously maintaining a safe profile [90]. The in silico ADMET prediction is a particularly efficient and cost-effective approach for initial screening of candidate compounds before proceeding to an expensive clinical trial phase [91]. Our study revealed that rheediachromenoxanthone **(5)** did not violate RO5, indicating its favorable drug-like properties. Additionally, it shows a high likelihood of HIA, comparable to chloroquine and artesunate. Drugs intended for non-CNS indications should avoid BBB permeation to prevent potential adverse effects on the CNS [92]. Our findings demonstrated that only chloroquine exhibited BBB permeability, which has previously been reported to possibly cause adverse effects such as headache, dizziness, vomiting, and psychosis [93]. In contrast, neither rheediachromenoxanthone **(5)** nor artesunate permeated the BBB, suggesting a lower risk of CNS-related adverse effects. All compounds were non-substrate for P-gp, suggesting that these compounds are prone to low efflux, enhancing their potential therapeutic efficacy. Rheediachromenoxanthone **(5)** inhibited several CYP enzymes but not CYP2C19, suggesting a lower likelihood of DDIs or associated adverse effects. The synthetic accessibility of compounds significantly affects drug design, particularly due to the challenges posed by the synthesis of certain compounds [94]. Rheediachromenoxanthone showed a low synthetic accessibility score, indicating ease of synthesis, thereby enabling the potential for its large-scale production. Toxicological assessment demonstrated that rheediachromenoxanthone **(5)** had minimal acute oral toxicity with a high predicted LD_50_ value. In addition, rheediachromenoxanthone **(5)** exhibited non-hepatotoxic, non-carcinogenic, and non-cytotoxic properties, enhancing its safety profile.

## Conclusions

Rheediachromenoxanthone, a pyranoxanthone compound isolated from the roots of *M. ferrea* L., exhibits good in vitro antiplasmodial activity while maintaining its non-toxicity towards normal cells (Vero cells). In addition, this compound exhibited a strong affinity for *Pf*LDH, favorable drug-like properties, good pharmacokinetic properties, ease of synthesis, and low toxicity. Based on our findings, rheediachromenoxanthone shows high potential for use as a scaffold in the design of novel antimalarial drugs targeting *Pf*LDH. Nevertheless, it is imperative to validate the potential of rheediachromenoxanthone through additional experimental investigations. These studies should include a medicinal chemistry-based approach to chemically modify the rheediachromenoxanthone scaffold to improve its antiplasmodial activities and pharmacokinetic properties. Furthermore, in vivo model studies are essential for a comprehensive validation of both the antimalarial efficacy and safety profiles. These efforts will advance the potential of rheediachromenoxanthone as a clinically effective antimalarial drug.

### Electronic supplementary material

Below is the link to the electronic supplementary material.

Supplementary Material 1

## Data Availability

No datasets were generated or analysed during the current study.

## Abbreviations

ACTs: Artemisinin-based combination therapies
ADMET: Absorption, Distribution, Metabolism, Excretion, and Toxicity
ADT: AutoDockTools
ATP: Adenosine triphosphate
BBB: Blood-brain barrier
CADD: Computer-aided drug design
CC: Column chromatography
CC_50_: The 50% cytotoxic concentration
CNS: Central nervous system
CYP: Cytochromes P450
DDIs: Drug-drug interactions
DHODH: Dihydroorotate dehydrogenase
DMSO: Dimethyl sulfoxide
DNA: Deoxyribonucleic acid
dTMP: Deoxythymidine monophosphate
FBS: Fetal bovine serum
GHS: Globally harmonized system of classification and labeling of chemicals
GMS: Greater Mekong Subregion
HBA: Hydrogen bond acceptors
HBD: Hydrogen bond donors
HIA: Human gastrointestinal absorption
hLDH: Human lactate dehydrogenase
IC_50_: The half maximal inhibitory concentration
LD_50_: Median lethal dose
NADH: 1,4-Dihydronicotinamide adenine dinucleotide
P-gp: Permeability glycoprotein
PDB: Protein Data Bank
PDBQT: Protein Data Bank, Partial Charge (Q), and Atom Type format
*Pf*DHFR-TS: *Plasmodium falciparum* dihydrofolate reductase-thymidylate synthase
*Pf*LDH: *Plasmodium falciparum* lactate dehydrogenase
pLDH: parasite lactate dehydrogenase
PLIP: Protein Ligand Interaction Profiler
QCC: Quick column chromatography
RMSD: Root Mean Square Deviation
RO5: Lipinski’s rule of five
SBDD: Structure-based drug design
SMILES: Simplified Molecular Input Line Entry System format
TLC: Thin-layer chromatography
TPSA: Topological polar surface area
