# Supplementary material for: Antiplasmodial potential of isolated xanthones from Mesua ferrea Linn. roots: an in vitro and in silico molecular docking and pharmacokinetics study
Source: BMC Complement Med Ther. 2024 Jul 25;24:282. doi: 10.1186/s12906-024-04580-5 (PMC11270968; doi:10.1186/s12906-024-04580-5)
Supplement: Supplementary file 1 — Supplementary Material 1 [file 12906_2024_4580_MOESM1_ESM.docx]

NMR spectroscopic data of compounds 1−7 isolated from *Mesua ferrea* L. roots

Table S1. NMR spectroscopic data of 1-hydroxy-7-methoxyxanthone (1)

| Position | Type of C | *δ*_H_ *^a^* (*J* in Hertz) | *δ*_C_ *^b^* | HMBC (^1^H→^13^C) |
| --- | --- | --- | --- | --- |
| 1 | C |  | 161.9 |  |
| 2 | CH | 6.78, *dd*, 8.4, 0.7 | 110.0 | C-4, C-9a |
| 3 | CH | 7.56, *t*, 8.4 | 136.4 | C-1, C-4a |
| 4 | CH | 6.91, *d*, 8.4 | 106.8 | C-2, C-4a, C-9a |
| 4a | C |  | 156.1 |  |
| 5 | CH | 7.39, *d*, 8.0 | 119.1 | C-7, C-9, C-10a |
| 6 | C | 7.34, *dd*, 8.0, 2.7 | 125.5 |  |
| 7 | C |  | 156.3 |  |
| 8 | CH | 7.60, *d*, 2.7 | 105.3 | C-6, C-9, C-9a |
| 8a | C |  | 120.9 |  |
| 9 | C |  | 181.9 |  |
| 9a | C |  | 108.7 |  |
| 10a | C |  | 151.0 |  |
| 1-OH |  | 12.66, *s* |  | C-1, C-2, C-9, C-9a |
| 7-OCH_3_ | CH_3_ | 3.91, *s* | 55.8 | C-7 |

*^a^* Recorded in 300 MHz

*^b^* Recorded in 75 MHz

Figure S1-1. Selected HMBC () and NOESY () correlations of 1-hydroxy-7-methoxyxanthone (1)


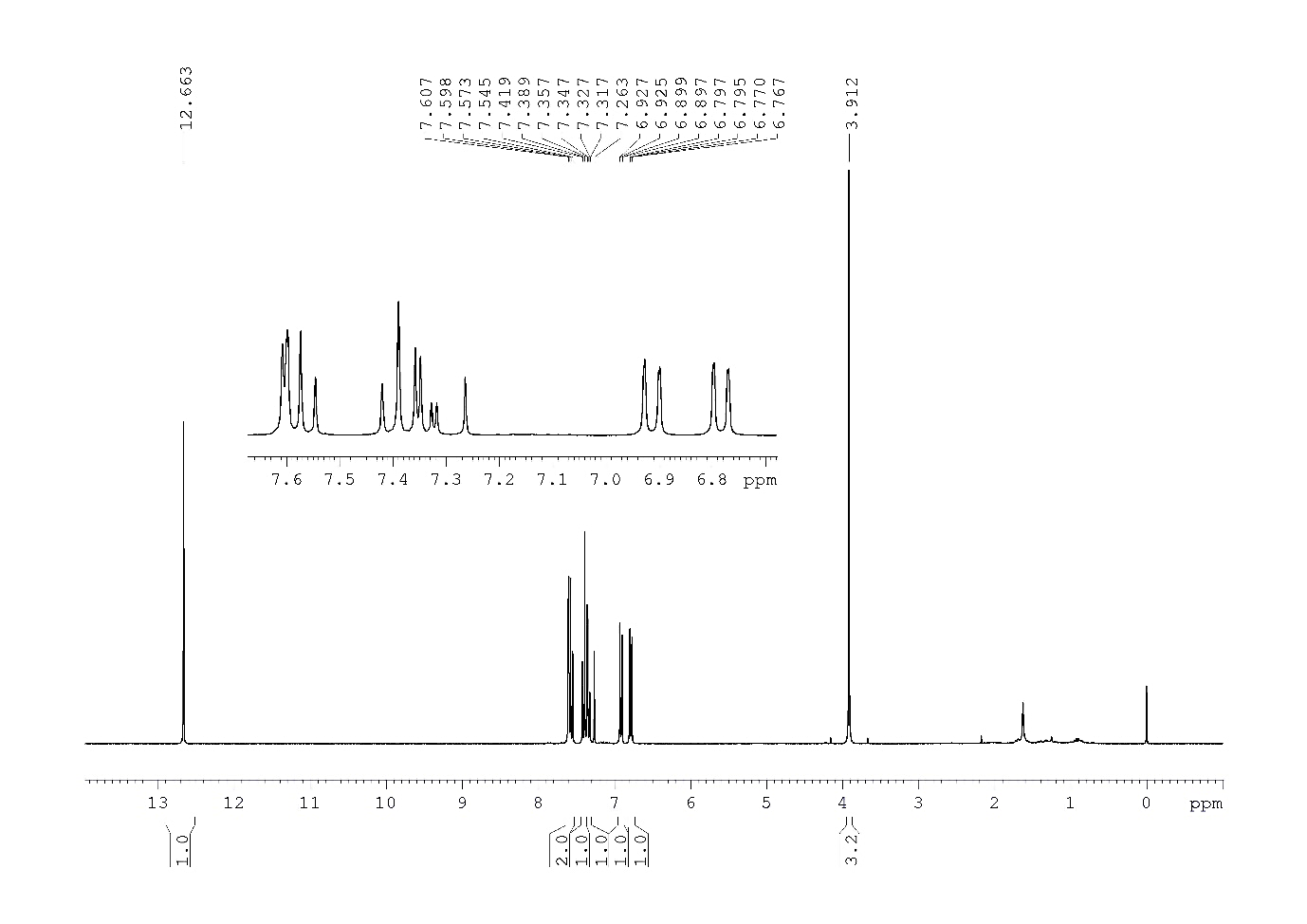


Figure S1-2. ^1^H NMR (300 MHz, CDCl_3_) spectrum of 1-hydroxy-7-methoxyxanthone (1)


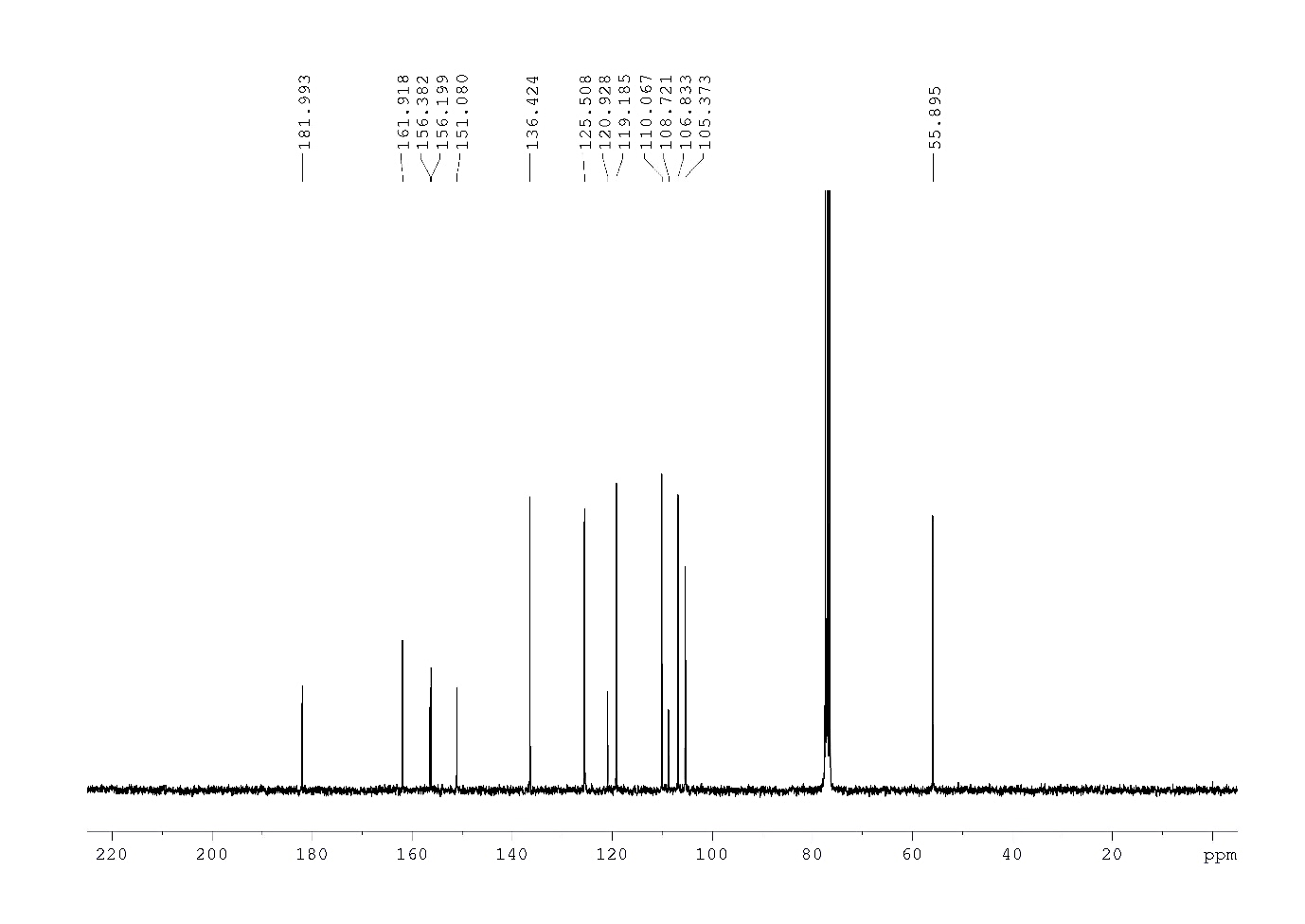


Figure S1-3. ^13^C NMR (75 MHz, CDCl_3_) spectrum of 1-hydroxy-7-methoxyxanthone (1)

Table S2. NMR spectroscopic data of 1-hydroxy-5-methoxyxanthone (2)

| Position | Type of C | *δ*_H_ *^a^* (*J* in Hertz) | *δ*_C_ *^b^* | HMBC (^1^H→^13^C) |
| --- | --- | --- | --- | --- |
| 1 | C |  | 161.8 |  |
| 2 | CH | 6.80, *dd*, 8.3, 1.2 | 110.4 | C-1, C-4, C-9a |
| 3 | CH | 7.59, *t*, 8.3 | 136.7 | C-1, C-4a |
| 4 | CH | 7.03, *dd*, 8.3, 1.2 | 107.2 | C-2, C-4a, C-9a |
| 4a | C |  | 156.0 |  |
| 5 | C |  | 148.4 |  |
| 6 | CH | 7.23, *dd*, 7.8, 1.8 | 116.0 | C-8, C-10a |
| 7 | CH | 7.30, *t*, 7.8 | 123.6 | C-5, C-8a |
| 8 | CH | 7.81, *dd*, 7.8, 1.8 | 116.7 | C-6, C-9 C-10a |
| 8a | C |  | 121.4 |  |
| 9 | C |  | 182.2 |  |
| 9a | C |  | 108.9 |  |
| 10a | C |  | 146.5 |  |
| 1-OH |  | 12.62, *s* |  | C-3, C-4a, C-9a |
| 5-OCH_3_ | CH_3_ | 4.02, *s* | 56.4 | C-5 |

*^a^* Recorded in 300 MHz

*^b^* Recorded in 75 MHz

Figure S2-1. Selected HMBC () and NOESY () correlations of 1-hydroxy-5-methoxyxanthone (2)


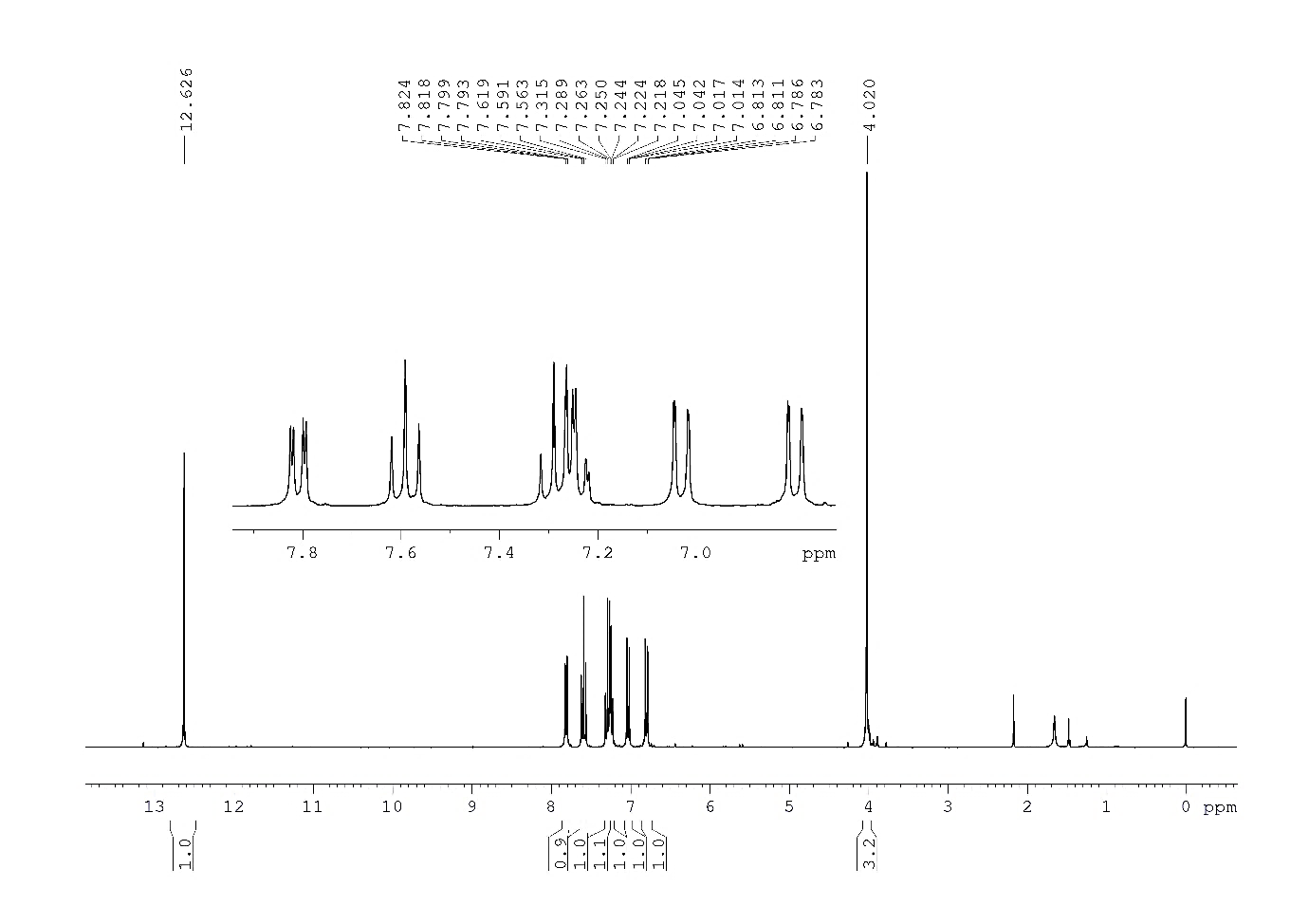


Figure S2-2. ^1^H NMR (300 MHz, CDCl_3_) spectrum of 1-hydroxy-5-methoxyxanthone (2)


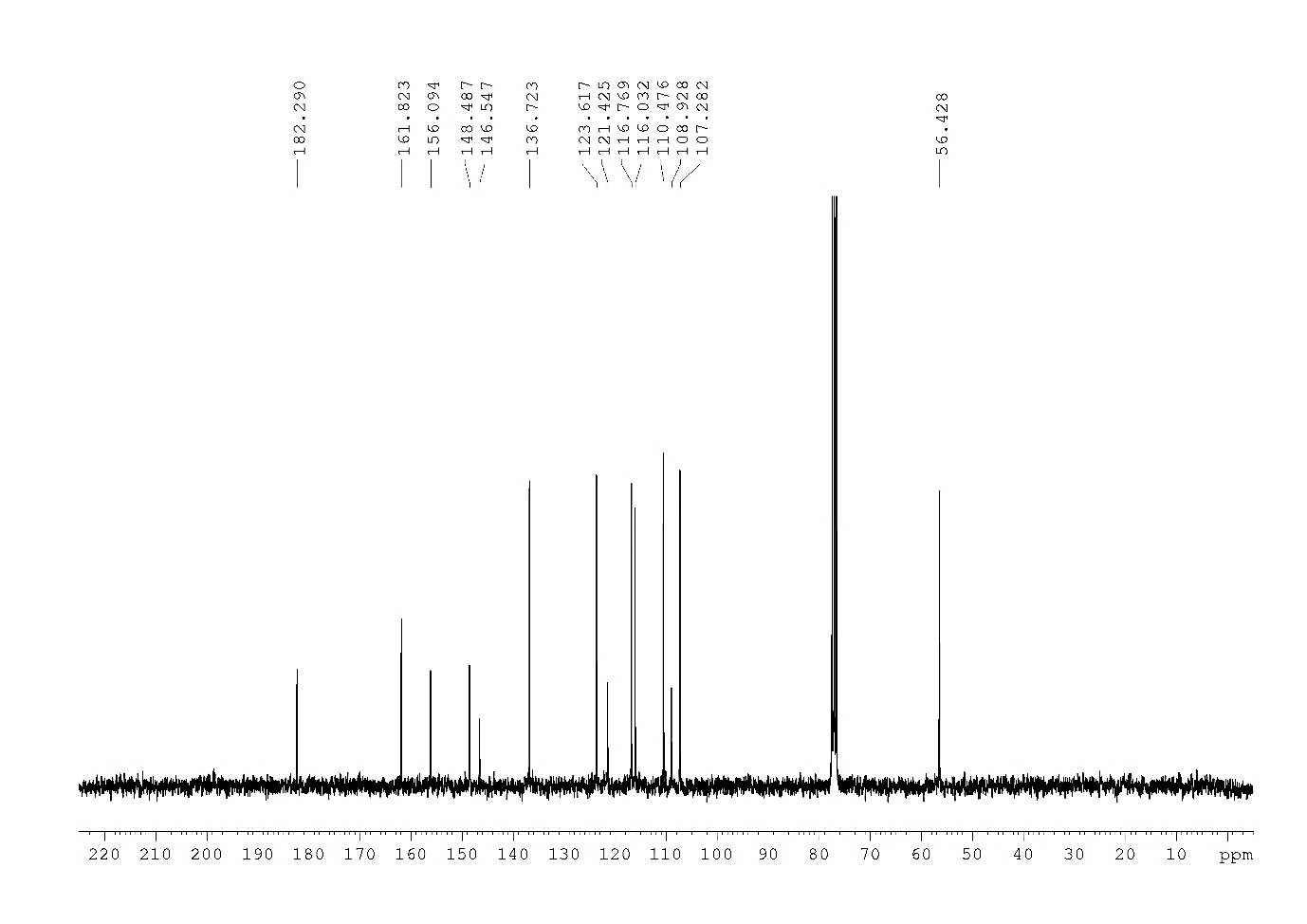


Figure S2-3. ^13^C NMR (75 MHz, CDCl_3_) spectrum of 1-hydroxy-5-methoxyxanthone (2)

Table S3. NMR spectroscopic data of 1,6-dihydroxyxanthone (3)

| Position | Type of C | *δ*_H_ *^a^* (*J* in Hertz) | *δ*_C_ *^b^* | HMBC (^1^H→^13^C) |
| --- | --- | --- | --- | --- |
| 1 | C |  | 162.3 |  |
| 2 | CH | 6.80, *d*, 8.0 | 109.6 | C-4, C-9a |
| 3 | CH | 7.97, *t*, 8.0 | 136.8 | C-1, C-4a |
| 4 | CH | 7.00, *d*, 8.0 | 106.8 | C-2, C-4a, C-9a |
| 4a | C |  | 156.4 |  |
| 5 | CH | 7.60, *d*, 3.0 | 108.3 | C-6, C-7, C-9 |
| 6 | C |  | 150.2 |  |
| 7 | CH | 7.44, *dd*, 9.0, 3.0 | 125.3 | C-5, C-6, C-8a |
| 8 | CH | 7.52, *d*, 9.0 | 119.2 | C-6, C-9, C-10a |
| 8a | C |  | 120.9 |  |
| 9 | C |  | 182.1 |  |
| 9a | C |  | 109.0 |  |
| 10a | C |  | 154.1 |  |
| 1-OH |  | 12.70, *s* |  | C-1, C-2, C-9, C-9a |
| 6-OH |  | 9.15, *br s* |  |  |

*^a^* Recorded in 500 MHz

*^b^* Recorded in 125 MHz

Figure S3-1. Selected HMBC () correlations of 1,6-dihydroxyxanthone (3)


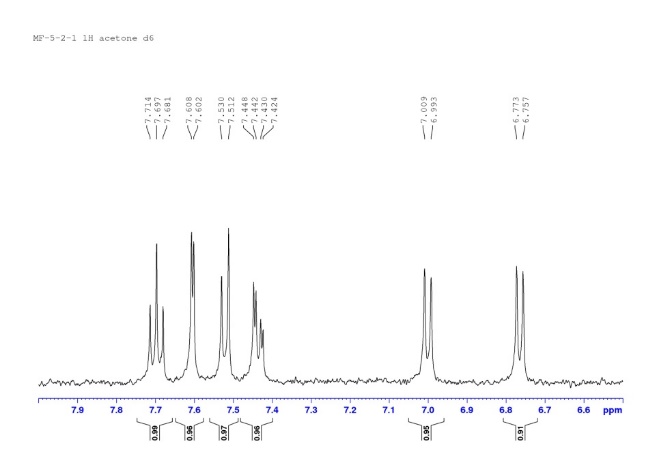

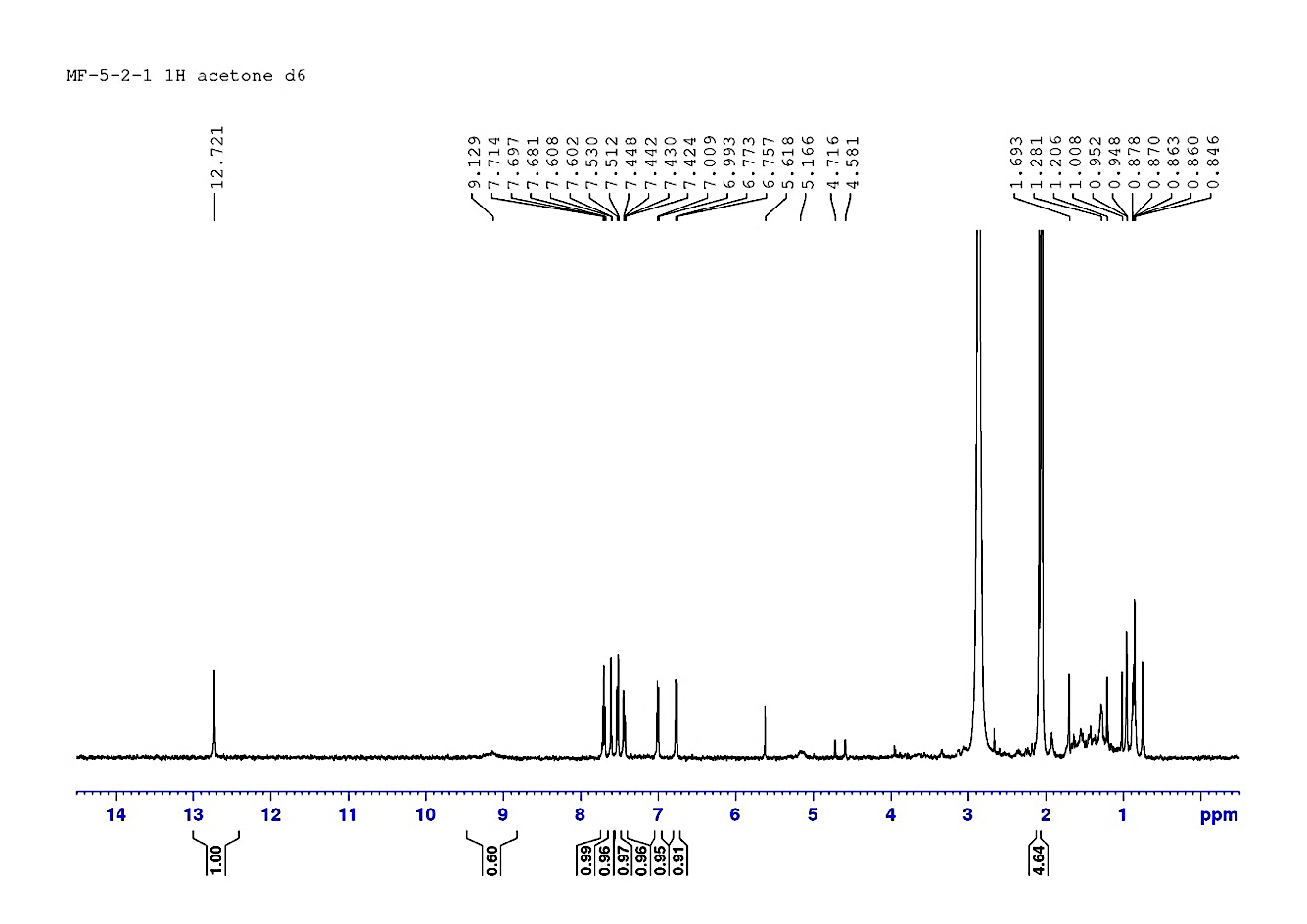


Figure S3-2. ^1^H NMR (500 MHz, acetone-*d*_6_) spectrum of 1,6-dihydroxyxanthone (3)


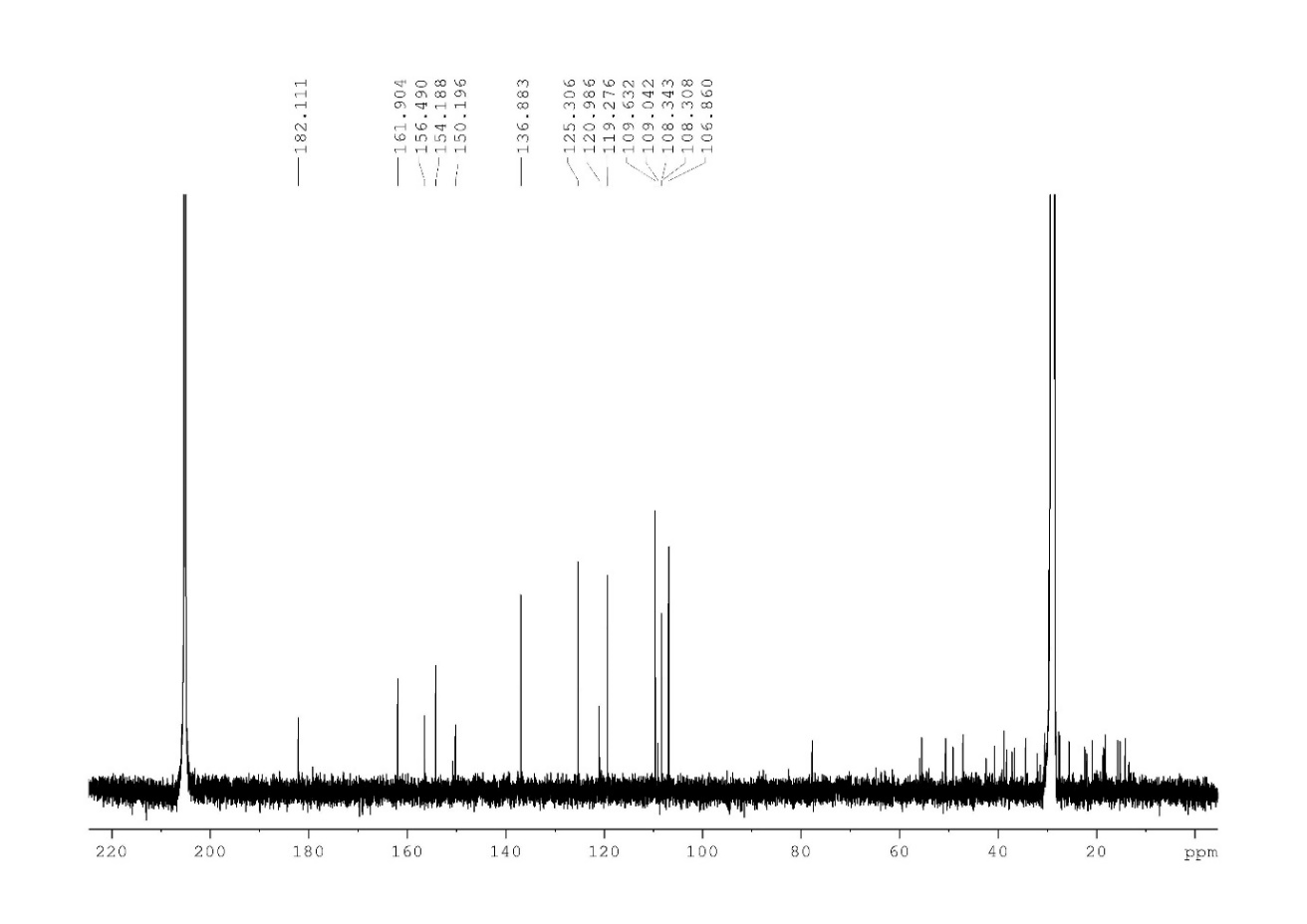


Figure S3-3. ^13^C NMR (125 MHz, acetone-*d*_6_) spectrum of 1,6-dihydroxyxanthone (3)

Table S4. NMR spectroscopic data of 1,5-dihydroxyxanthone (4)

| Position | Type of C | *δ*_H_ *^a^* (*J* in Hertz) | *δ*_C_ *^b^* | HMBC (^1^H→^13^C) |
| --- | --- | --- | --- | --- |
| 1 | C |  | 162.0 |  |
| 2 | CH | 6.82, *d*, 8.4 | 110.0 | C-1, C-4, C-9a |
| 3 | CH | 7.75, *t*, 8.4 | 136.9 | C-1, C-4a |
| 4 | CH | 7.05, *d*, 8.4 | 106.9 | C-2, C-4a, C-9a |
| 4a | C |  | 156.1 |  |
| 5 | C |  | 146.2 |  |
| 6 | CH | 7.75, *dd*, 7.8, 1.7 | 115.5 | C-8, C-10a |
| 7 | CH | 7.30, *t*, 7.8 | 124.1 | C-5, C-8a |
| 8 | CH | 7.74, *dd*, 7.8, 1.7 | 121.0 | C-6, C-9 C-10a |
| 8a | C |  | 121.3 |  |
| 9 | C |  | 182.4 |  |
| 9a | C |  | 108.5 |  |
| 10a | C |  | 145.5 |  |
| 1-OH |  | 12.73, *s* |  | C-3, C-4a, C-9a |
| 5-OH |  | 9.50, *br s* |  | C-5 |

*^a^* Recorded in 300 MHz

*^b^* Recorded in 75 MHz

Figure S4-1. Selected HMBC () and NOESY () correlations of 1,5-dihydroxyxanthone (4)


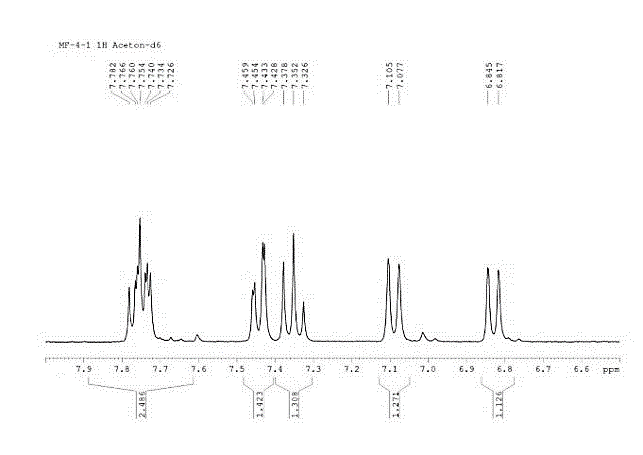

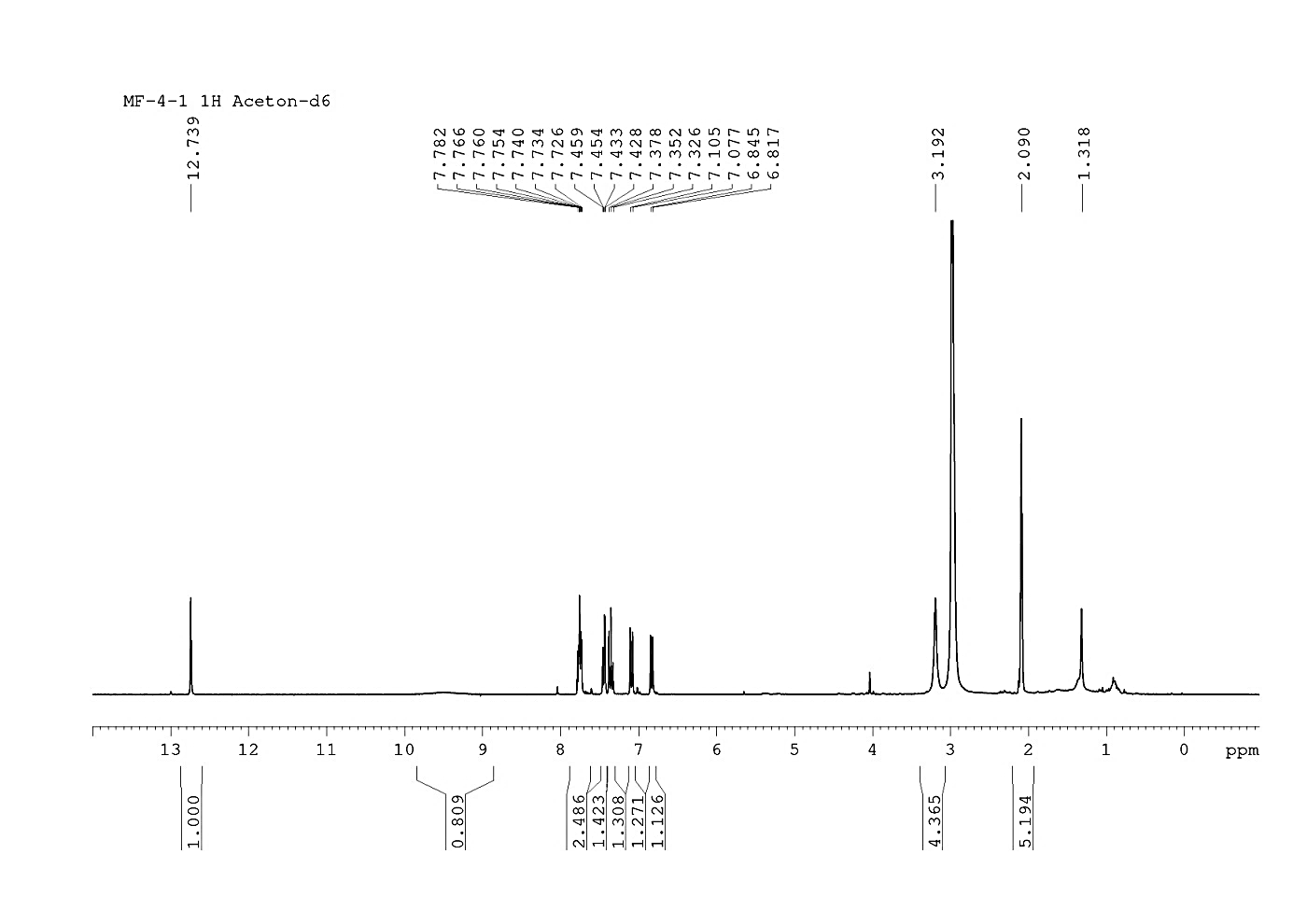


Figure S4-2. ^1^H NMR (300 MHz, acetone-*d*_6_) spectrum of 1,5-dihydroxyxanthone (4)


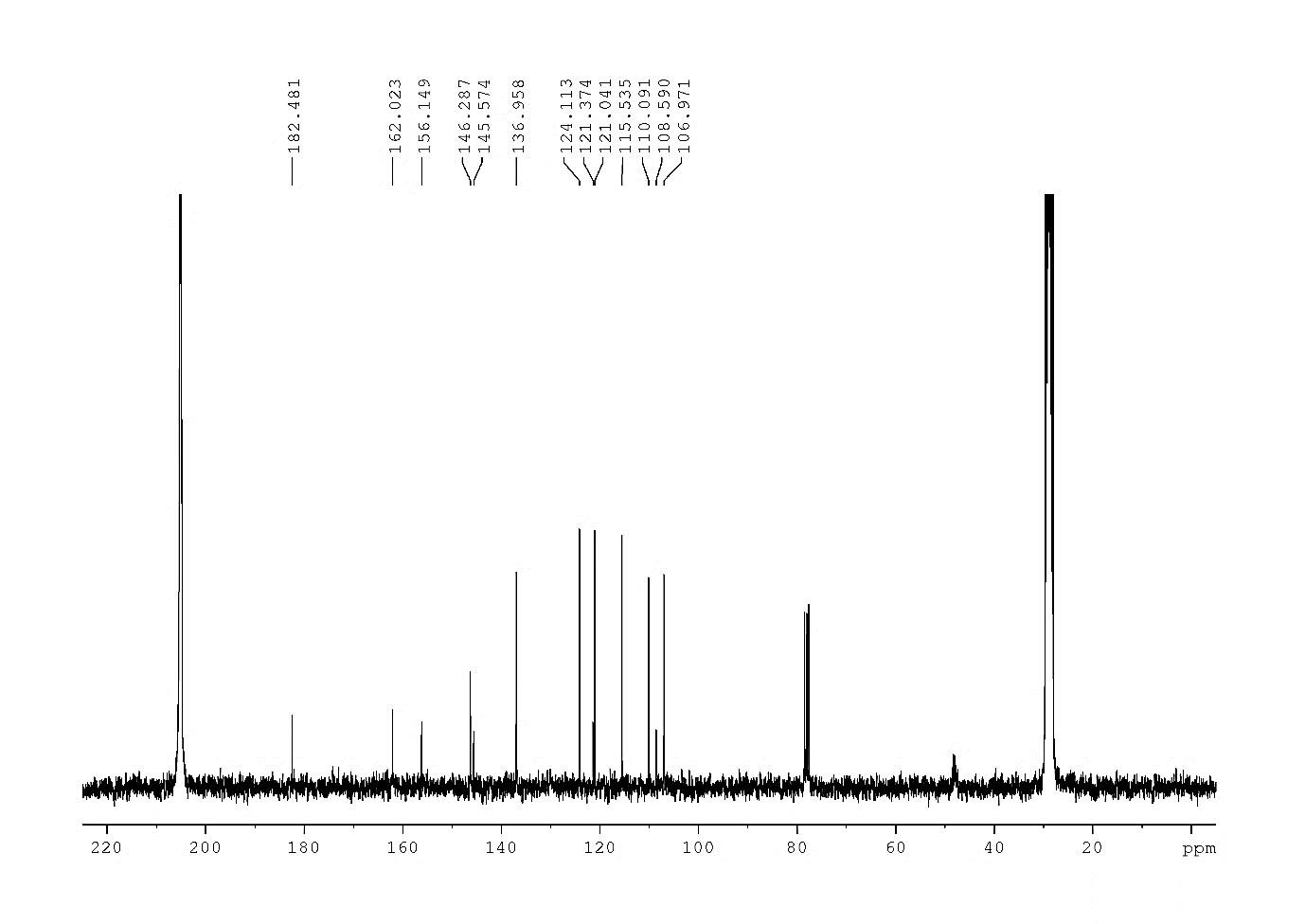


Figure S4-3. ^13^C NMR (75 MHz, acetone-*d*_6_) spectrum of 1,5-dihydroxyxanthone (4)

Table S5. NMR spectroscopic data of rheediachromenoxanthone (5)

| Position | Type of C | *δ*_H_ *^a^* (*J* in Hertz) | *δ*_C_ *^b^* | HMBC (^1^H→^13^C) |
| --- | --- | --- | --- | --- |
| 1 | C |  | 162.0 |  |
| 2 | CH | 6.75, *d*, 8.5 | 110.0 | C-1, C-4, C-9a |
| 3 | CH | 7.68, *t*, 8.5 | 136.3 | C-1, C-4a |
| 4 | CH | 7.01, *d*, 8.5 | 106.8 | C-2, C-4a, C-9, C-9a |
| 4a | C |  | 156.2 |  |
| 5 | C |  | 133.4 |  |
| 6 | C |  | 146.2 |  |
| 7 | C |  | 118.6 |  |
| 8 | CH | 7.48, *s* | 112.5 | C-6, C-7, C-9, C-1′ |
| 8a | C |  | 114.5 |  |
| 9 | C |  | 181.5 |  |
| 9a | C |  | 108.2 |  |
| 10a | C |  | 156.2 |  |
| 1′ | CH | 6.60, *d*, 10.0 | 121.1 | C-6, C-7, C-8, C-3′ |
| 2′ | CH | 5.93, *d*, 10.0 | 131.7 | C-1′, C-3′, C-4′, C-5′ |
| 3′ | C |  | 72.1 |  |
| 4′ | CH_3_ | 1.50, *s* | 27.6 | C-6, C-2′, C-3′ |
| 5′ | CH_3_ | 1.50, *s* | 27.6 | C-6, C-2′, C-3′ |
| 1-OH |  | 12.89, *s* |  | C-1, C-2, C-9 |
| 5-OH |  |  |  |  |

*^a^* Recorded in 500 MHz

*^b^* Recorded in 125 MHz

Figure S5-1. Selected HMBC () and NOESY () correlations of rheediachromenoxanthone (5)


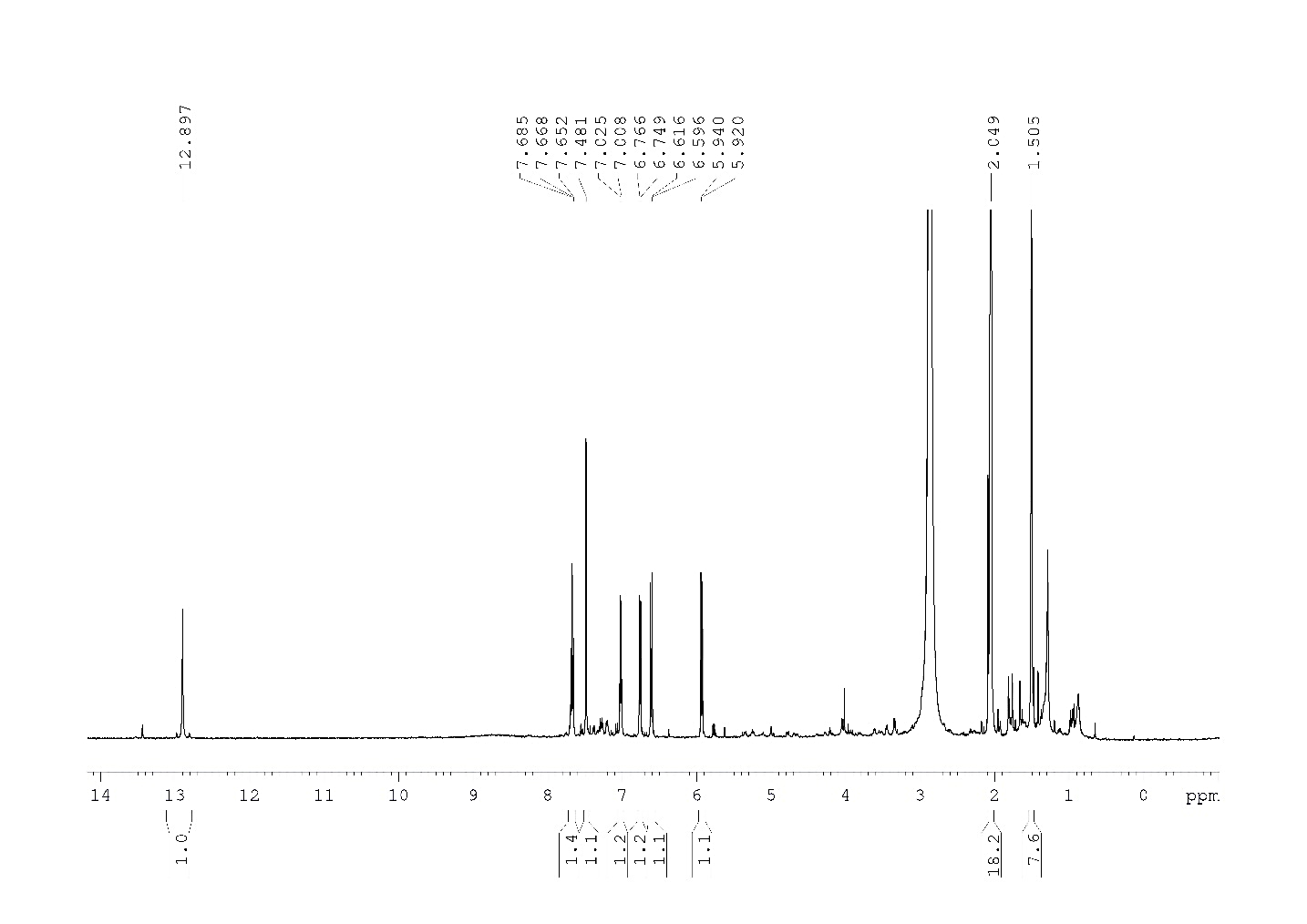


Figure S5-2. ^1^H NMR (500 MHz, acetone-*d*_6_) spectrum of rheediachromenoxanthone (5)


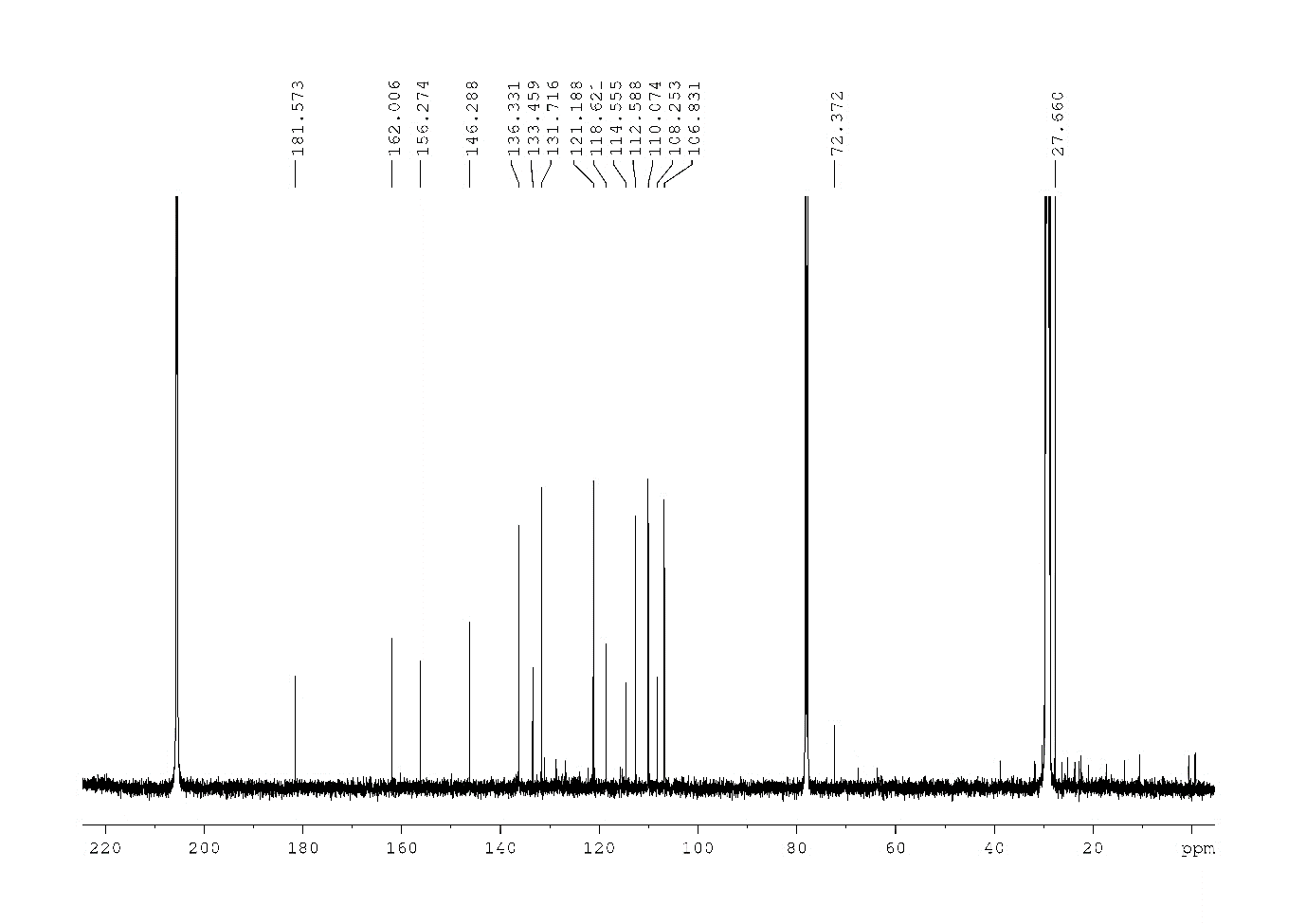


Figure S5-3. ^13^C NMR (125 MHz, acetone-*d*_6_) spectrum of rheediachromenoxanthone (5)

Table S6. NMR spectroscopic data of 1,5-dihydroxy-3-methoxyxanthone (6)

| Position | Type of C | *δ*_H_ *^a^* (*J* in Hertz) | *δ*_C_ *^b^* | HMBC (^1^H→^13^C) |
| --- | --- | --- | --- | --- |
| 1 | C |  | 163.5 |  |
| 2 | CH | 6.37, *d*, 2.1 | 96.9 | C-1, C-3, C-4, C-9a |
| 3 | C |  | 167.0 |  |
| 4 | CH | 6.58, *d*, 2.1 | 92.4 | C-2, C-3, C-4a, C-9a |
| 4a | C |  | 157.5 |  |
| 5 | C |  | 146.1 |  |
| 6 | CH | 7.38, *dd*, 7.8, 1.8 | 120.6 | C-8, C10a |
| 7 | C | 7.30, *t*, 7.8 | 124.1 | C-5, C-8a |
| 8 | CH | 7.68, *dd*, 7.8, 1.8 | 115.3 | C-6, C-9, C-10a |
| 8a | C |  | 121.3 |  |
| 9 | C |  | 180.9 |  |
| 9a | C |  | 103.3 |  |
| 10a | C |  | 145.2 |  |
| 1-OH |  | 12.93, *s* |  | C-1, C-2, C-3, C-9a |
| 3-OCH_3_ | CH_3_ | 4.00, *s* | 55.5 | C-3 |
| 5-OH |  | 9.40, *br s* |  |  |

*^a^* Recorded in 300 MHz

*^b^* Recorded in 75 MHz

Figure S6-1. Selected HMBC () and NOESY () correlations of 1,5-dihydroxy-3-methoxyxanthone (6)


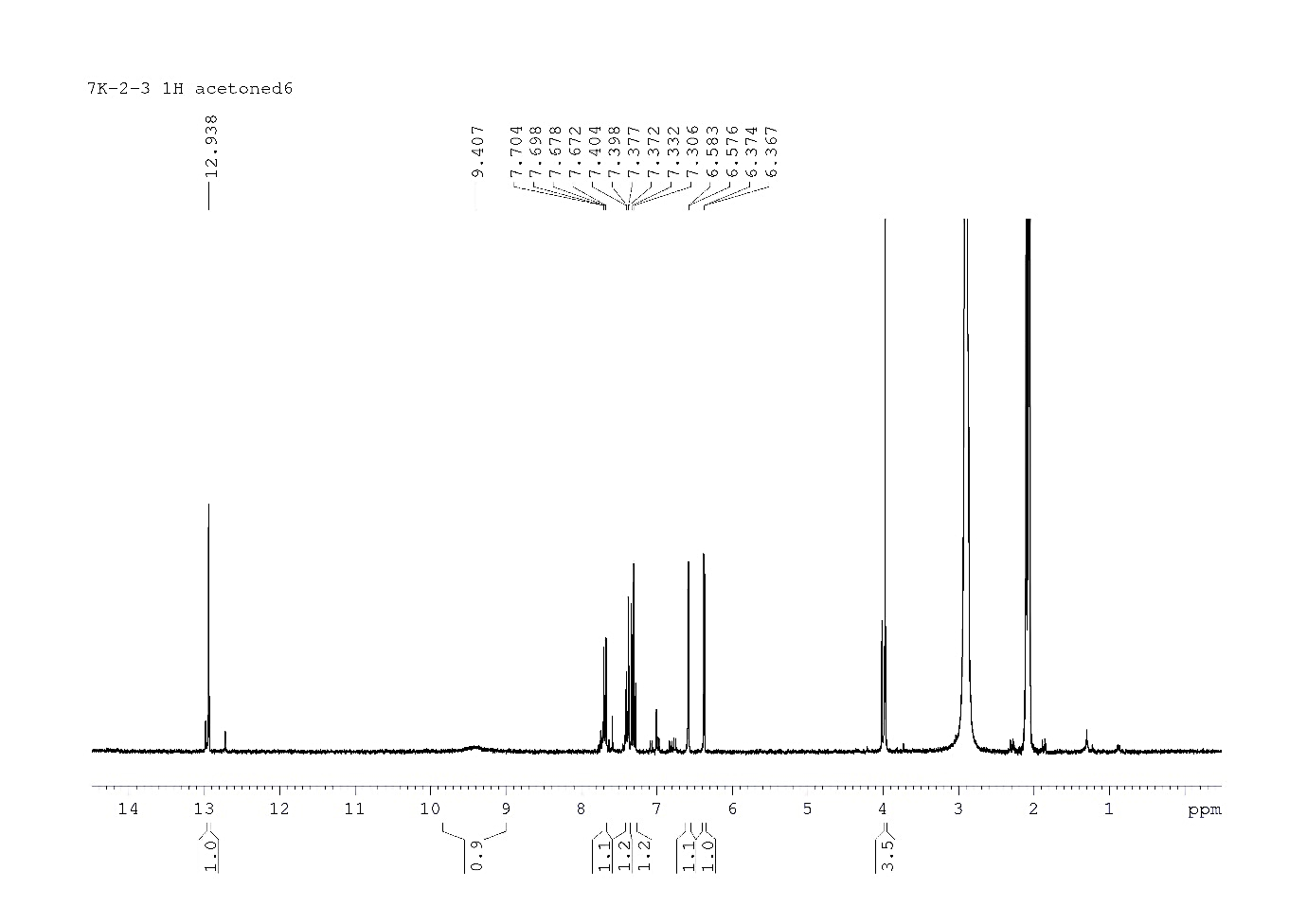


4.002

Figure S6-2. ^1^H NMR (300 MHz, acetone-*d*_6_) spectrum of 1,5-dihydroxy-3-methoxyxanthone (6)


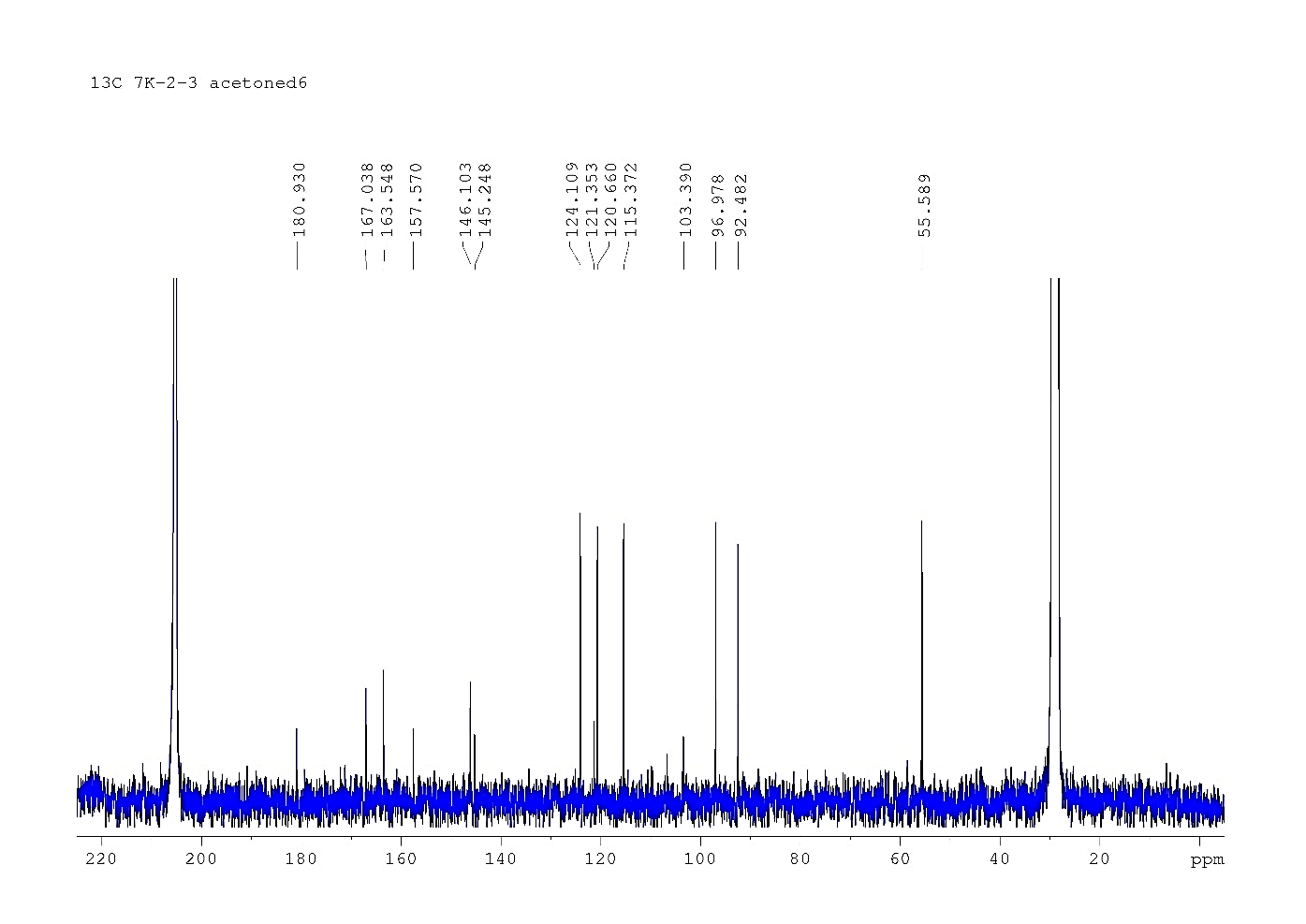


Figure S6-3. ^13^C NMR (75 MHz, acetone-*d*_6_) spectrum of 1,5-dihydroxy-3-methoxyxanthone (6)

Table S7. NMR spectroscopic data of 2,5-dihydroxy-1-methoxyxanthone (7)

| Position | Type of C | *δ*_H_ *^a^* (*J* in Hertz) | *δ*_C_ *^b^* | HMBC (^1^H→^13^C) |
| --- | --- | --- | --- | --- |
| 1 | C |  | 145.2 |  |
| 2 | C |  | 146.6 |  |
| 3 | CH | 7.42, *d*, 9.0 | 123.0 | C-1, C-2, C-4a |
| 4 | CH | 7.30, *d*, 9.0 | 113.7 | C-2, C-9a |
| 4a | C |  | 150.2 |  |
| 5 | C |  | 146.0 |  |
| 6 | CH | 7.29, *dd*, 8.1, 1.8 | 119.3 | C-8, C-10a |
| 7 | CH | 7.22, *t,* 8.1 | 123.3 | C-5, C-6, C-8a |
| 8 | CH | 7.69, *dd*, 8.1, 1.8 | 116.0 | C-6, C-9 C-10a |
| 8a | C |  | 122.9 |  |
| 9 | C |  | 175.4 |  |
| 9a | C |  | 116.2 |  |
| 10a | C |  | 144.7 |  |
| 1-OCH_3_ | CH_3_ | 3.93, *s* | 61.2 | C-1 |
| 2-OH |  | 9.20, *br s* |  | C-2 |
| 5-OH |  | 8.33, *br s* |  | C-5 |

*^a^* Recorded in 300 MHz

*^b^* Recorded in 75 MHz

Figure S7-1. Selected HMBC () and NOESY () correlations of 2,5-dihydroxy-1-methoxyxanthone (7)


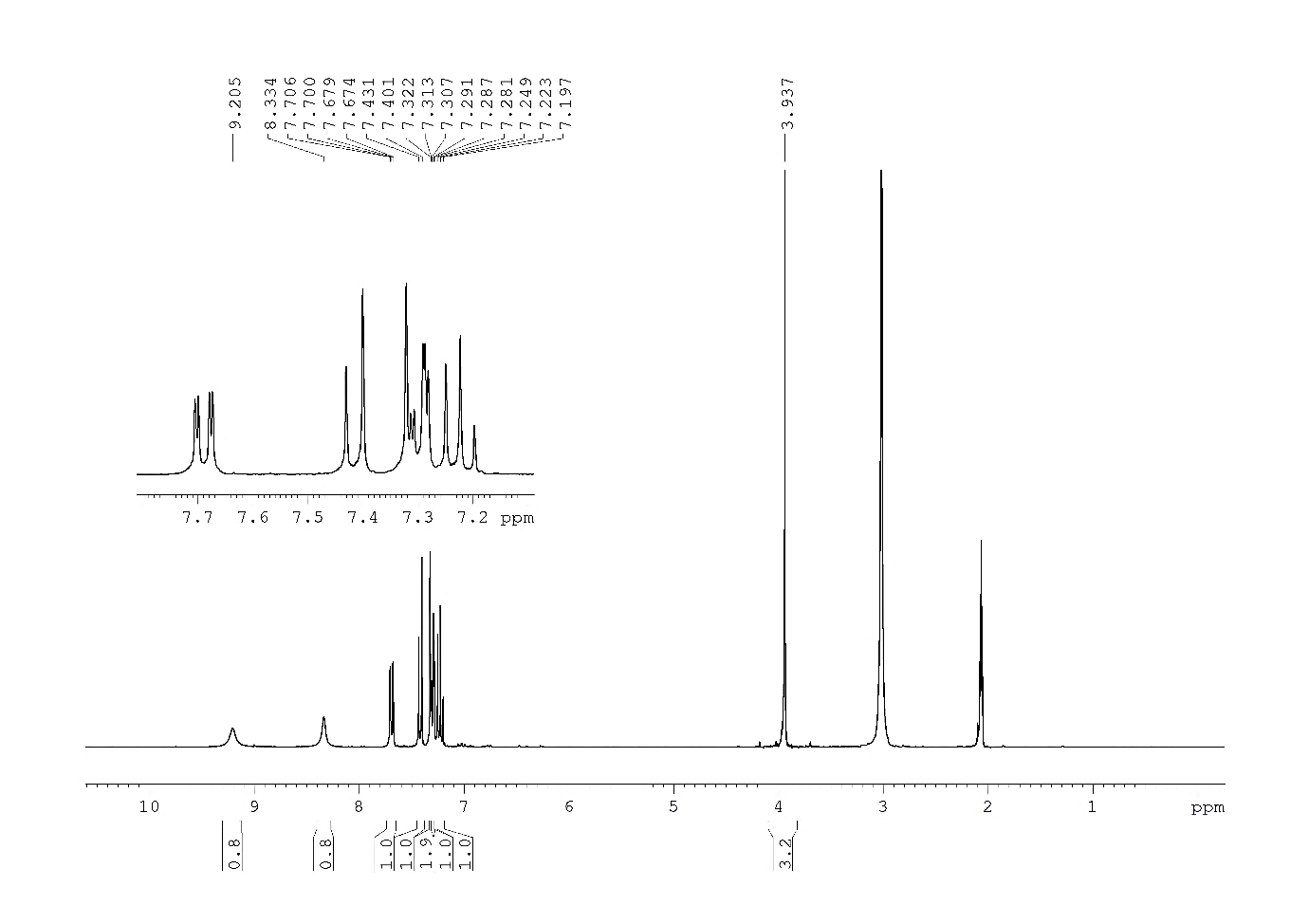


Table S7-2. ^1^H NMR (300 MHz, acetone-*d*_6_) spectrum of 2,5-dihydroxy-1-methoxyxanthone (7)


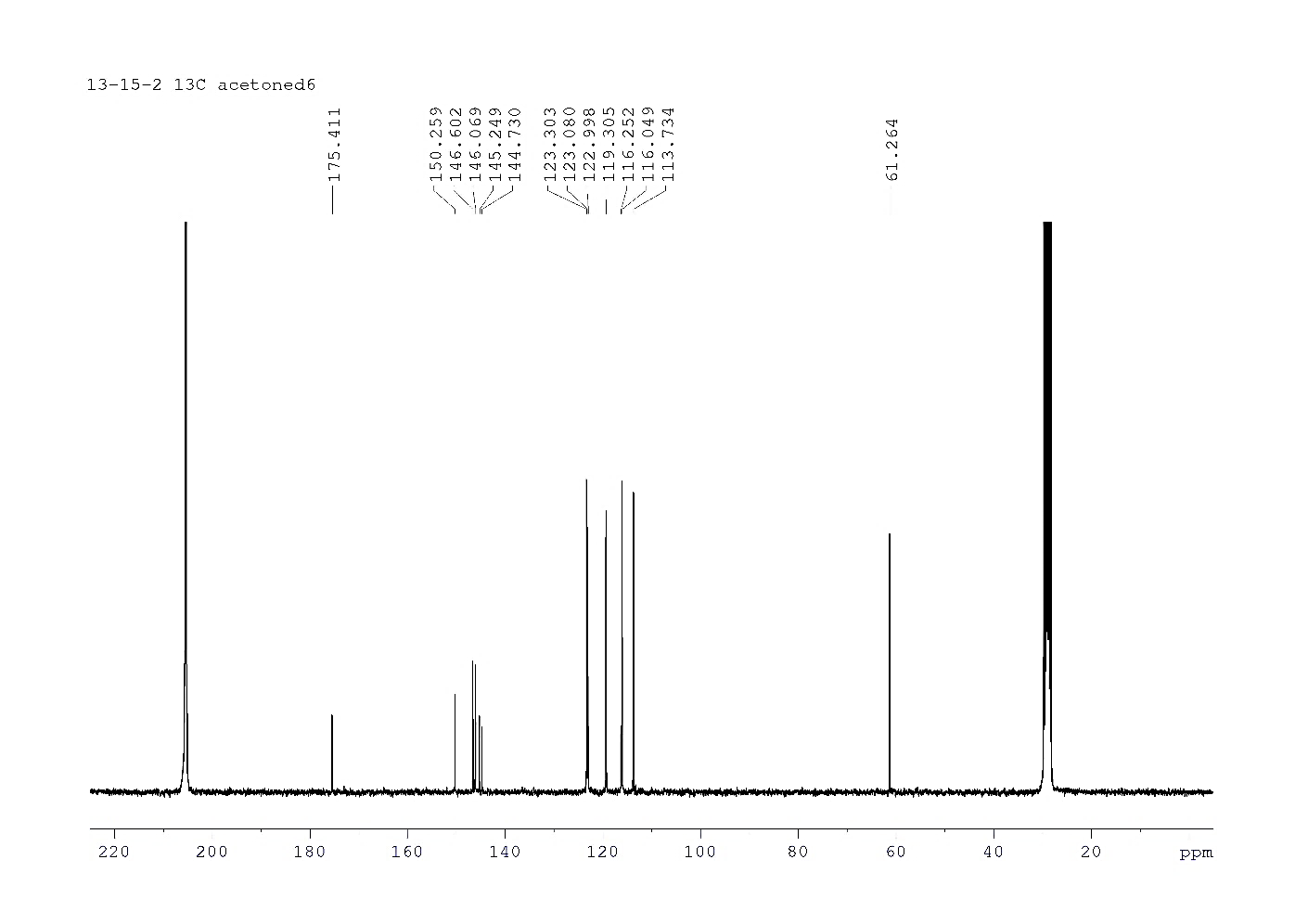


Table S7-3. ^13^C NMR (75 MHz, acetone-*d*_6_) spectrum of 2,5-dihydroxy-1-methoxyxanthone (7)

Reference

Chukaew, A. (2019). Chemical constituents from the roots and twigs of *Mesua ferrea* L. [Unpublished Ph.D.’s thesis]. Prince of Songkla University.
